# Non-viral CRISPR activation system targeting VEGF-A and TGF-β1 for enhanced osteogenesis of pre-osteoblasts implanted with dual-crosslinked hydrogel

**DOI:** 10.1016/j.mtbio.2022.100356

**Published:** 2022-07-11

**Authors:** Guo Chen, Shaohui Deng, Mingxiang Zuo, Jin Wang, Du Cheng, Bin Chen

**Affiliations:** a Key Laboratory for Polymeric Composite & Functional Materials of Ministry of Education, School of Materials Science and Engineering, Sun Yat-sen University, Guangzhou, 510275, PR China; b Division of Orthopaedics and Traumatology, Department of Orthopaedics, Nanfang Hospital, Southern Medical University, Guangzhou, 510515, PR China; c Department of Radiology, The Third Affiliated Hospital of Sun Yat-sen University, Guangzhou, 510630, PR China

**Keywords:** Bone regeneration, Nanomaterials for gene delivery, CRISPR activation, Dual cross-linked hydrogel

## Abstract

Healing of large calvarial bone defects remains challenge but may be improved by stimulating bone regeneration of implanted cells. The aim of this study is to specially co-activate transforming growth factor β1 (TGF-β1) and vascular endothelial growth factor (VEGF-A) genes expressions in pre-osteoblast MC3T3-E1 cells through the non-viral CRISPR activation (CRISPRa) system to promote osteogenesis. A cationic copolymer carrying nucleus localizing peptides and proton sponge groups dimethyl-histidine was synthesized to deliver CRISPRa system into MC3T3-E1 cells with high cellular uptake, lysosomal escape, and nuclear translocation, which activated VEGF-A and TGF-β1 genes expressions and thereby additively or synergistically induced several osteogenic genes expressions. A tunable dual-crosslinked hydrogel was developed to implant the above engineered cells into mice calvaria bone defect site to promote bone healing *in vivo*. The combination of multi-genes activation through non-viral CRISPRa system and tunable dual-crosslinked hydrogel provides a versatile strategy for promoting bone healing with synergistic effect.

## Highlights

• A cationic polymer carrying nucleus localizing peptides and proton sponge groups was used to deliver CRISPRa system.
• A tunable dual-crosslinked hydrogel was developed to implant the engineered cells into bone defect site*.*
• The combination of CRISPRa system and tunable crosslinked hydrogel provides a platform for bone healing treatment.

## Graphical abstract

**Figure:**
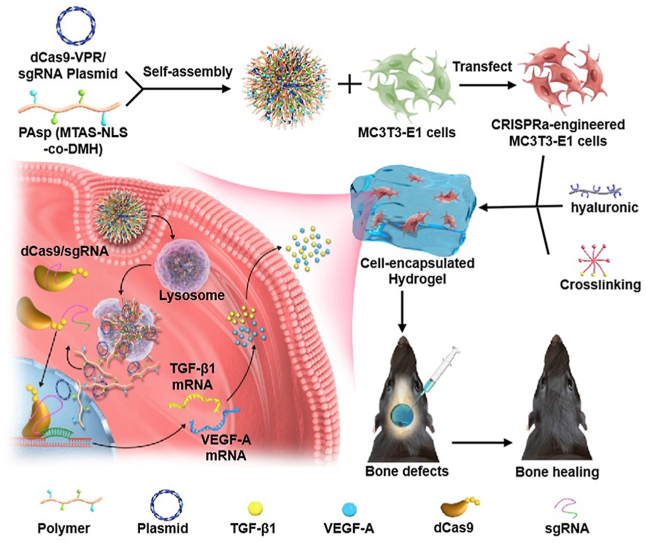

## CRediT author statement

**Guo Chen:** Conceptualization, Methodology, Writing original draft. **Shaohui Deng:** Writing-review & editing, Software, Data curation. **Mingxiang Zuo:** Formal analysis, Data curation. **Jin Wang:** Resources. **Du Cheng** & **Bin Chen:** Conceptualization, Writing-review & editing, Resources, Supervision, Project administration.

## 1 Introduction

Bone renewal is often compromised because of the limited regeneration capacity of bone self-healing particularly in critical-size bone defects, resulting in delayed union and nonunion fracture [1]. Autografts and allografts have been developed to resolve this dilemma with optimal skeletal incorporation [2]. However, their uses are limited by host morbidity and low availability of autografts and extrusion of allografts [3]. Thus, synthetic bone-grafts are required to remedy the shortages of natural bone implants. The synthetic bone-grafts harness three main elements including osteogenic cells, osteogenic factors, and structural scaffolds, to mimic the natural bone regeneration process in which the coordinated expression of multiple osteogenic factors plays a critical role [4,5]. Some growth factors act individually, while certain combinations of these factors promote bone regeneration synergistically [6]. During the bone regeneration process, osteogenic and angiogenic processes are closely connected [7]. Vascular endothelial growth factor-A (VEGF-A), member of the VEGF family, plays a critical role in angiogenesis [8], and transforming growth factor-beta 1 (TGF-β1) regulates osteoblast proliferation and differentiation during bone growth and development [9]. The TGF-β1 alone downregulates bone formation-related genes (e.g., alkaline phosphate (ALP) gene, osteocalcin (OC) gene, and collagen type II (COL-II) gene) expressions, but the combination of VEGF-A and TGF-β1 upregulates these genes expressions [10]. In addition, the combination of these two factors stimulates the secretion of bone morphogenetic proteins (BMPs) to regulate osteoblastic progenitor cells and pre-osteoblasts. Therefore, the combination of VEGF-A and TGF-β1 is a promising approach to enhance bone regeneration capacity.

To increase the contents of these factors, the direct protein delivery and gene transfer strategies have been used in multiple types of cells (e.g., Mesenchymal stem cell (MSC) and pre-osteoblast). Although protein delivery-based therapy shows substantial effect on bone regeneration, its clinical application faces several issues regarding bioactivity loss after incorporation into a scaffold, short life time, high cost, and high dose-induced systemic cytotoxicity [11]. The gene transfer persistently produces therapeutic protein in a physiological manner at ectopic sites, but may cause some unwanted changes of host gene structure. The CRISPR activation (CRISPRa) system activates gene expression by binding with transcription factors at normal physiological sites rather than at ectopic sites, through fusing a catalytically inactive Cas9 (dCas9) with transcription activators such as VP64 [12] or with a tripartite activator VPR [13] to induce specific expression of target gene by guiding with a single-guide RNA (sgRNA) [14]. The activation of *Sox9* gene by viral vector-based CRISPRa (viral CRISPRa) promotes the differentiation of BMSC into cartilage [15]. The co-activation of *Wnt10b* and *Foxc2* genes through viral CRISPRa in rat BMSCs enhanced osteoblast differentiation *in vitro* and significantly improved skull bone repair *in vivo* [16]. However, the clinical applications of the viral CRISPRa are hindered by several limitations including uncontrolled chromosomal integration, immunogenicity, and limited packaging capacity as well as scale-up production. By contrast, the non-viral CRISPRa has advantages including high safety, low immunogenicity, and enhanced genetic payloads.

To deliver the plasmid DNA (pDNA) encoding CRISPRa and CRISPR components into target cells, some cationic materials including polymers, liposomes, and inorganic materials have been used as carriers. Cationic liposomes [17] were used to encapsulate negatively charged pDNA encoding CRISPR-Cas9 system (CRISPR-Cas9 plasmid) through electrostatic interactions, repairing mutation of the *α-**l**-iduronidase* gene and disrupting the gene structure of *polo-like kinase 1* [18]. The CRISPR-Cas9 plasmids are co-precipitated with CaCO_3_ to knock out the *CTNNB1* gene encoding β-catenin [19]. Cationic polymers (e.g., PEI, dendrimer and helical peptide) are used to complex with CRISPR-Cas9 plasmids to form nanoparticles [20], enabling efficient gene editing. In addition, the hybrids of these materials are also developed to deliver CRISPR-Cas9 plasmids [21]. However, few non-viral vectors are prepared to activate target gene expression through non-viral delivery of CRISPR-dCas9 plasmids. To the best of our knowledge, only PEI-based non-viral vectors were explored to deliver CRISPR-dCas9 plasmids for activation of microRNA-524 *in vivo* [22]. To enhance the delivery efficiency of CRISPR-dCas9 plasmid, the non-viral vectors with lysosomal escape and nuclear translocation properties are required. To avoid the degradation of pDNA in lysosome, some groups with tertiary amines including dimethyl-histidine (DMH), 2-(diisopropylamino) ethylamine (DIP), and polyethyleneimine (PEI) were introduced into non-viral carriers [23]. The fused peptide of nuclear localization sequence (NLS) and microtubule associated sequence (MTAS) was used to transport pDNA into the nucleus [24]. Thus, the DMH and NLS-MTAS peptide-based nanocarrier may be an effective platform to deliver CRISPRa system targeting *VEGF-A* and *TGF-β1* genes into osteoblast.

The scaffold should not only encapsulate the engineered cells but also offer a suitable environment for bone regeneration through mimicking natural extracellular matrix [25]. Hyaluronic acid (HA)-based hydrogels are attracting attention due to their high-water content, high viscoelastic nature, and high permeability for exchange of nutrients and metabolites [26]. Moreover, several types of cross-linking reactions including Michael additions, Diels-Alder (DA) reactions, azide-alkyne cycloadditions, and condensation reactions between carbonyl groups and N-nucleophiles were combined to optimize the mechanical strength, gelation rate, and degradation rate of HA hydrogel [27]. For instance, the combination of DA click chemistry and acyl-hydrazone bond formation resulted in a multifunctional HA hydrogel with excellent mechanical properties [28]. Two types of HA (i.e., aldehyde- and ketone-modified HA) with different reaction rates with poly(ethylene glycol) (PEG)-oxyamine were jointly used to achieve controllable cell encapsulation by regulating the gelation rate of HA hydrogels [29]. The crosslinking reaction between aminooxy and aldehyde groups has been widely used to prepare injectable hydrogels due to the high reactivity and mild reaction conditions [30], but its gelation rate is limited by low second-order rate constants commonly below 0.1 ​M^−1^ ​s^−1^ [31]. Thus, the combination of the slow-reacting aldehyde-amine Schiff-base and fast-reacting cycloaddition-based reactions is an alternative approach to control hydrogel properties including gelation rate, mechanical strength, and degradation rate. Besides, a particular target of hydrogels is the development of an easily prepared injectable hydrogel with in situ gelation behavior after injection in a minimal injure [32].

**Fig. 1:**
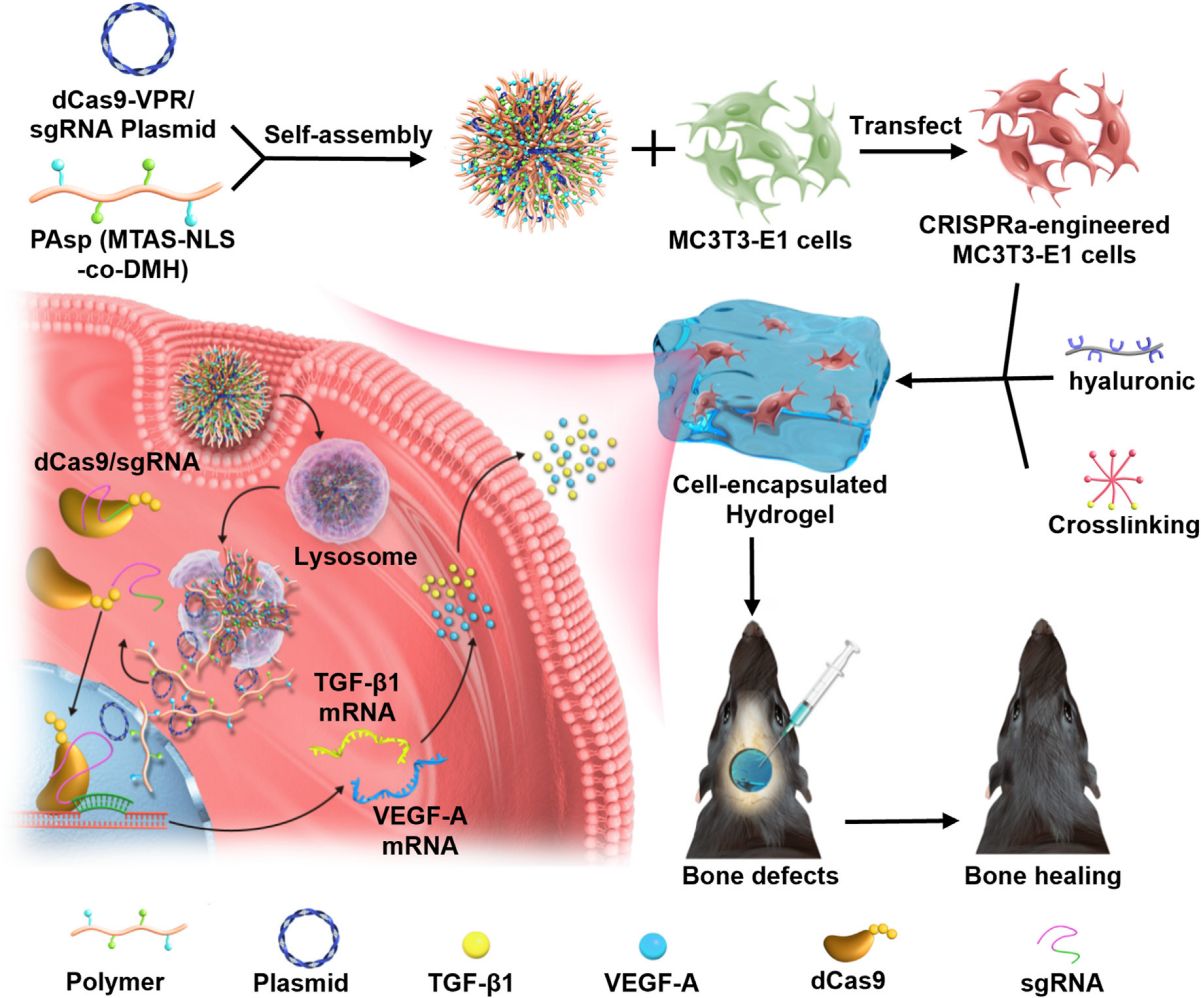
Schematic of the preparation of non-viral CRISPRa system and intracellular co-activation of VEGF-A and TGF-β1 for bone healing. Cationic polymers PAsp (MTAS-NLS-*co*-DMH) complex with plasmids encoding CRISPRa system into nanoparticles; after internalization by MC3T3-E1 cells, pH-sensitive groups DMH rapture lysosome allowing release of nanoparticles into cytoplasm through proton sponge effect; nuclear localizing peptides NLS-MTAS guid plasmid into nucleus to express mRNAs encoding dCas9 protein and sgRNA, which specially activates the expressions of VEGF-A and TGF-β1; the non-viral CRISPRa system-engineered pre-osteoblasts MC3T3-E1 are implanted with dual-crosslinked hyaluronic acid-based hydrogel to repair calvarial defect.

In this study, we prepared a pH-sensitive cationic polymer decorated with nucleus localizing peptide NLS-MTAS to complex CRISPRa plasmid targeting *VEGF-A* and *TGF-β1* genes (Fig. 1). The pre-osteoblast MC3T3-E1 cells effectively internalized the nanocomplexes, resulting in significant activation of *VEGF-A* and *TGF-β1*, which promoted the osteoblastic activity and mineralization. A dual-crosslinked HA hydrogel formed by Schiff-base and azide-alkyne cycloadditions reactions was fabricated to encapsulate CRISPRa system-engineered MC3T3-E1 in an injectable manner. The bone regeneration capacity of the hydrogel was investigated both *in vitro* and *in vivo*.

## 2 Materials and methods

### 2.1 Materials

Plasmids SP-dCas9-VPR (#63798) and phU6-gRNA (#53188) were purchased from Addgene (Cambridge, USA). pcDNA3.1+C-EGFP (pEGFP) was purchased from GenScript Biotechnology (Nanjing, China). Lipofectamine™ 2000, 3-(4,5-dimethylthiahiazol-2-yl)-2,5-diphenyl-2H-tetrazolium bromide (MTT), LysoTracker™ Deep Red, DND-26, 4’,6-diamidino-2-phenylindole (DAPI), Hoechst 33,342, and YOYO™-1 Iodide (491/509) (YOYO-1) were purchased from Invitrogen (Gaithersburg, MD, USA). Heparin, NaN_3_, nocodazole, cytochalasin-D (CD), amiloride, dynasore, chlorpromazine (CPZ), and nystatin were purchased from Sigma-Aldrich (St. Louis, MO, USA). All other chemicals for the synthesis of polymers were purchased from Sigma-Aldrich unless otherwise specified. The MTAS-NLS peptide (GRYLTQETNKVETYKEQPLKTPGKKKKGKPGKRKEQEKKKRRTR) and control peptide (scrambled MTAS-NLS peptide) were synthesized by the Haode Peptide company (Wuhan, China).

Mouse osteoblast precursor MC3T3-E1 cells were purchased from the Cell Bank of the Chinese Science Academy (Shanghai, China). MC3T3-E1 cells were cultured in alpha-MEM medium (Corning) with 10% fetal bovine serum and 1% penicillin and streptomycin (100 IU/mL). Cells were sub-cultured using 0.25% trypsin (Gibco) containing ethylene diaminetetraacetic acid after reaching 90% confluence. All reagents and consumables for cell culture were purchased from Gibco (Life Technologies). To induce osteogenesis, osteogenic medium containing 50 ​μg/mL l-ascorbic acid (Sigma-Aldrich), 10 ​mM β-glycerophosphate (Sigma-Aldrich), and 10 ​nm dexamethasone (Sigma-Aldrich) was added. The medium and osteogenic factors were changed every 2 days.

### 2.2 Preparation and characterization of polyaspartate(NLS-MTAS-co-DMH)/pDNA nanoparticles (PND/pDNA-NPs)

After the cationic copolymer polyaspartate(NLS-MTAS-co-DMH), refereed to here as PND, was dissolved in sodium acetate/acetate (NaAc/HAc) buffer solution (PBS, pH 5.5), pDNA solutions (0.2 ​mg/mL in water) were added into the polymer solution at predetermined amounts according to N/P ratios. Here, N/P ratios indicate the moles of nitrogen (N) in the polymer PND relative to the moles of phosphate groups (P) in the pDNA. For example, 6 ​μg of PND polymer containing 30.98 ​nmol of N and 1 ​μg of pDNA containing 3.07 ​nmol of P were used to prepare PND/pDNA-NPs with an N/P ratio of 10/1. The mixture was vigorously ultrasonicated for 30 ​s and kept at room temperature for 30 ​min to form polyplexes referred to here as PND/pDNA-NPs.

### 2.3 Characterization of polymer and nanoparticle

^1^H NMR spectra were measured with a 400 ​MHz spectrometer (Bruker, Germany) using DMSO-*d*_6_ at room temperature. For transmission electron microscopy (TEM) analysis of PND/pDNA-NPs, 5 ​μL of PND/pDNA-NPs (0.1 ​mg/mL) was dropped to a copper grid coated with amorphous carbon. After being dried at 40 ​°C, the sample was stained with a phosphotungstic acid solution (3 ​wt%) and observed using a transmission electron microscope (JEOL JEM-1400 Plus, Japan) at 120 ​kV. Sizes and zeta potentials of the PND/pDNA-NPs were measured by dynamic light scattering (DLS) at 25 ​°C (Malvern NANO ZS, UK). Polyplexes between the polymer PND and pDNA were evaluated by gel retardation assays. The solutions containing PND/pDNA-NPs were stained with GoldenView (NEB, Beverly, MA, USA) and different volumes at a concentration of 100 ​ng DNA/well were loaded into 1% agarose gels. The electrophoresis was performed under 100 ​V for 30 ​min using a Bio-Rad Sub-Cell electrophoresis cell (Bio-Rad Laboratories, Inc., US) and images were obtained with a DNR Bio-Imaging System (Jerusalem, Israel) under UV light.

### 2.4 Cytotoxicity assay

The cytotoxicity of PND/pDNA-NPs was evaluated via an MTT [3-(4,5-dimethylthiazol-2-yl)-2,5-diphenyltetrazolium] kit according to the manufacturer's instructions. Cells (1 ​× ​10^4^) were cultured in a 96-well plate and incubated for 24 ​h. Various concentrations of PAsp(NLS-MTAS-*co*-DMH) were then added to the cells. The cell viability was analyzed using the MTT assay.

### 2.5 Cellular uptake

Cationic polymers PAsp(NLS-MTAS-*co*-DMH) were used to self-assemble with plasmid encoding enhanced green fluorescence protein (pEGFP) into nanoparticles PND/pEGFP-NPs. Briefly, cells were inoculated at a density of 5 ​× ​10^4^ ​cells per well in 12-well plates and cultured for 12 ​h. Nanoparticles were prepared at various N/P ratios (i.e., 6, 8, 10, 12, 14, 16), respectively. The nanoparticles were added to the wells and then incubated with cells under the serum-free α-MEM at 37 ​°C for 4 ​h. Afterward, the medium was replaced with serum-containing medium. Cells were further incubated for 48 ​h. Cells transfected with pEGFP using Lipofectamine™ 2000 were used as the control group. Cells were washed three times with PBS solution and detached with 0.25% trypsin solution. After being resuspended in 500 ​μL PBS solution, at least 5 ​× ​10^4^ live cells of each group were subjected to flow cytometry analysis. The EGFP-positive cells were quantified using flow cytometry (Life Technology, Carlsbad, USA) at 25 ​°C using a 530/30 ​nm band pass filter. All statistics were collected in triplicate and three independent tests were carried out. The transfection efficiency was expressed as percentage of EGFP-positive cells.

### 2.6 Intercellular distribution

Cells were plated in 35-mm culture dishes at a density of 1 ​× ​10^6^ ​cells per dish and incubated for 24 ​h at 37 ​°C under a humidified atmosphere with 5% CO_2_. The pDNA was stained with YOYO-1 dyes. Cell nuclei and lysosomes were stained with Hoechst 33342 and LysoTracker™ Deep Red, respectively. Fluorescence of nucleus, pDNA, and lysosome were observed with CLSM at different time points (e.g., 2 ​h, 4 ​h, and 6 ​h) after addition of PND/pDNA-NPs. The intracellular distribution of dCas9-VPR protein was recorded through immunofluorescence (IF) analysis. Briefly, MC3T3-E1 cells were transfected with PND/pDNA-NPs. After incubation with PND/pDNA-NPs for 4 ​h, the culture medium containing PND/pDNA-NPs was replaced with fresh medium plus FBS. IF analyses of dCas9-VPR protein were performed at different time points post-transfection. After being washed three times with PBS, cells were fixed for 10 ​min in 4% paraformaldehyde and permeabilized with 0.15% Triton X-100 for 15 ​min. Cells were washed with PBS three times for 5 ​min and incubated with 1% BSA in PBST (PBS plus 0.1% Tween 20) for 1 ​h. Cells were incubated with a 200 ​× ​dilution of anti-CRISPR-Cas9 antibody (ab191468, Abcam, Cambridge, UK) in PBST plus 1% BSA in a humidified chamber overnight at 4 ​°C. Cells were washed with PBS three times for 5 ​min and incubated with a fluorescence-labeled secondary antibody (ab150115, Cambridge, UK) for 1 ​h at room temperature. All operations were performed in the dark. Cells were observed with confocal laser scanning microscope (CLSM, Leica SP8, Germany) to determine cellular distribution of pDNA and dCas9-VPR protein.

### 2.7 Construction and identification of sgRNA plasmid (psgRNA) targeting TGF-β1 and VEGF-A genes

Candidate sgRNAs were designed using the online CRISPR Design Tool (crispr.mit.edu) to be complementary to sequences between −800 and +100 bp from the transcription start sites of the *TGF-β1* and *VEGF-A* genes. Ten pairs of 25-bp complementary DNA oligonucleotides with a 20-bp target sequence were synthesized by Sangon Biotech (Shanghai, China) as templates for sgRNA transcriptions. Two complementary oligonucleotides were annealed to generate a double-stranded DNA (dsDNA) with 4-bp overhangs and cloned into *Bbs*I-digested phU6-sgRNA to generate psgRNA plasmids.

A mixture of pdCas9-VPR and psgRNA plasmids at a weight ratio of 1:1 was used to prepare nanopolyplexes PND/pDNA-NPs. MC3T3-E1 cells were plated in 12-well plates at a density of 5 ​× ​10^4^ ​cells/well and cultured for 12 ​h. The cells were transfected with PND/pDNA-NPs at a concentration of 1 ​μg/mL DNA per well. After incubation at 37 ​°C for 48 ​h, TGF-β1 and VEGF-A mRNA levels were quantified using RT-PCR method with SYBR Green Master Mix. Total RNA was extracted from cells using TRIzol Reagent (Invitrogen, CA, USA). The total RNA was then reverse transcribed into cDNA using the PrimeScript RT Master Mix Kit (Takara, Dalian, China) and amplified using the LightCycle® 480 SYBR Green I Master Kit (Roche, Switzerland). The PCR amplification was carried out for 40 cycles under the following conditions: 95 ​°C for 10 ​min; 95 ​°C for 15 ​s; 60 ​°C for 1 ​min; and 72 ​°C for 30 ​s using the Step One plus Real-time PCR System (ABI, USA). Mouse *GAPDH* gene was used as the internal reference. The primers for specific genes (*TGF-β1* and *VEGF-A* genome sequences, osteogenic genes of bone sialoprotein (BSP), osteopontin (OPN) and Collagen Type I Alpha 1 (COL1a1)) and the internal reference (mouse GAPDH) were prepared by Sangon Biotech company (Shanghai, China) (Table S2). All experiments were conducted in triplicate.

### 2.8 Protein expression assay

MC3T3-E1 cells were harvested 48 ​h after transfection. Total proteins were isolated from lysed cells using the RIPA lysis buffer (Abcam, Cambridge, UK) in the presence of protease inhibitors. Total protein content was determined using a bicinchoninic acid (BCA) protein assay kit (Sangon Biotech Co. Ltd., Shanghai, China), 30 ​μg proteins were electrophoresed in 10% SDS-PAGE and transferred onto a PVDF membrane (Millipore Corp., USA). After incubation with 5% (w/v) BSA (MP, Auckland, New Zealand) for 1 ​h, the membranes were probed with primary antibodies (Abcam, Cambridge, UK) overnight at 4 ​°C. The membranes were immersed into solutions containing secondary antibodies (Abcam, Cambridge, UK) for 1 ​h. The protein bands were visualized with a luminol reagent (Santa Cruz Biotechnology, Dallas, Texas, USA) in a chemiluminescence imaging system (GE Image Quant LAS 500, USA). TGF-β1 (Quantikine ELISA Kit, cat# F11590, Westang) and VEGF-A (Quantikine ELISA Kit, cat# F11669, Westang) levels in cell supernatants were determined with ELISA kits according to the manufacturer's instructions. Color changes caused by avidin-horseradish peroxidase-mediated reactions were monitored by measuring optical absorbance at 450 ​nm.

### 2.9 *In vitro* osteogenesis analysis

MC3T3-E1 cells were plated in 6-well plates at a density of 5 ​× ​10^5^ ​cells/well and cultured for 12 ​h. The cells were transfected with PND/T-NPs, PND/V-NPs, and PND/TV-NPs at a concentration of 1 ​μg/mL DNA per well. After incubation at 37 ​°C for 6 ​h, the medium was replaced by osteogenic medium. The osteogenic medium was changed every other day. For ALP staining, at days 7, 14, and 28 after transfection, the cells were washed twice with PBS and fixed in 4% paraformaldehyde for 15 ​min, followed by incubation with BCIP/NBT ALP Color Development Kit (Beyotime, China) at 37 ​°C for several minutes. The staining reaction was stopped by PBS wash until the ALP-positive cells stained blue. Digital images of cells were captured with a microscope (Leica DM750, Germany). ALP activity was measured using an ALP assay kit (Beyotime, China), following the manufacturer's protocol. The results were normalized to the total intracellular protein content determined by the BCA Protein Assay Kit (Beyotime, China) and expressed in nanomoles of produced *p*-nitrophenol per min per mg of protein (nmol/(min·mg) protein).

Alizarin Red staining was performed at 14 ​d and 28 ​d after transfection to evaluate calcium deposition. Cells were washed with PBS, fixed in 4% paraformaldehyde for 30 ​min at room temperature, and then washed with deionized water (dH_2_O) twice. Extracellular calcium was stained by incubating the cell layers in the Alizarin Red S (Sigma, St. Louis, MO, USA) solution saturated in dH_2_O (pH 4.1). Nonspecific staining was removed by washing the wells with dH_2_O five times under gentle agitation. For quantification of ARS staining, CPS area were scanned using a desktop scanner, and the area fraction of the test areas were determined with ImageJ (Version 2.0.0) as described [33].

### 2.10 Preparation of aldehyde hyaluronic acid (HA-CHO)

2 ​g (0.0002 ​mmol) of hyaluronic acid (HA) were added into a 500-mL reaction flask, and fully dissolved in 250 ​mL dH_2_O under nitrogen-gas protection for 20 ​min, followed by the addition of 0.013 ​g (0.06 ​mmol) of sodium periodate. The reaction was performed in an ice bath for 48 ​h. Subsequently, 490 ​μL of ethylene glycol aqueous solution (10% v/v) was added to terminate the reaction. The solution was dialyzed (MWCO: 14 ​KDa) against NaCl aqueous solution (0.1 ​mol/L) for 3 ​d and continuously dialyzed against dH_2_O for 4 ​d. After that, the reaction solution was lyophilized to obtain 1.38 ​g of white powdery solid (HA-CHO).

### 2.11 Preparation of azido/cyclooctyne modified PEG

After 0.008 ​g (0.05 ​mmol) of *p*-azidobenzoic acid was dissolved in 1.5 ​mL of DMSO, 0.024 ​g (0.125 ​mmol) of 1-(3-dimethylaminopropyl) -3-ethylcarbodiimide hydrochloride (EDCI), and 0.014 ​g (0.125 ​mmol) of N-hydroxysuccinimide (NHS) were added. The reaction mixture was kept being stirred at room temperature overnight. An aliquot of 0.2 ​g (0.01 ​mmol) 8-arm polyethylene glycol amino (8 Arm–NH_2_–PEG) dissolved in 5 ​mL dH_2_O was added. The reaction was performed at room temperature for 24 ​h. Subsequently, the solution was dialyzed (MWCO: 7 ​KDa) against dH_2_O for 48 ​h and lyophilized. Finally, a light-yellow solid azido-modified PEG (N_3_-PEG) was obtained.

An aliquot of 0.02 ​g (0.05 ​mmol) dibenzocyclooctyne-succinimide ester (DBCO-NHS) in 4 ​mL DMSO and 0.2 ​g 8-arm polyethylene glycol amino (8-arm–NH_2_–PEG) (0.01 ​mmol) in 5 ​mL dH_2_O were mixed in a 25-mL reaction flask. The reaction mixture was kept being stirred at room temperature for 12 ​h. The reaction solution was subsequently dialyzed (MWCO: 7 ​KDa) against dH_2_O for 48 ​h and then lyophilized. Finally, a white solid cyclooctyne-modified PEG (DBCO-PEG) was obtained.

### 2.12 Preparation of dual-crosslinked hydrogels

0.01 ​g of HA-CHO and 0.015 ​g of 8-arm–NH_2_–PEG were dissolved in 120 ​μL of dH_2_O and allowed to stand for 30 ​min to obtain a light-yellow liquid (HA-PEG-hydrazone). Afterward, 0.0025 ​g of N_3_-PEG dissolved in the dH_2_O was added to the HA-PEG-hydrazone and followed with standing for 30 ​min at room temperature to obtain a yellow-brown viscous liquid. The product was denoted as HA-N_3_. The DBCO-modified HA (HA-DBCO) can be obtained using the same method. Briefly, 0.0025 ​g of DBCO-PEG dissolved in 30 ​μL of dH_2_O was added to HA-PEG-hydrazone and allowed to stand for 30 ​min at room temperature. Finally, a yellow viscous liquid HA-DBCO was obtained. The mentioned liquid HA-N_3_ and HA-DBCO were mixed in a 4-mL centrifuge tube and incubated in a water bath at 37 ​°C. Finally, a gel-like solid was obtained. This dual-crosslinked HA hydrogel was named as HA-HS hydrogel.

### 2.13 Gelation time of samples

The gelation behavior was determined using the tube inversion method. Liquids HA-N_3_ and HA-DBCO were prepared at different mass ratios of HA-CHO to NH_2_-PEG to N_3_-PEG/DBCO-PEG in a serum bottle at 37 ​°C according to the method described in Section 2.10.3. The formation of hydrogels was evaluated according to the reported protocol [34]. The gelation time was determined as the time from initiating mixing of the polymer solution to the formation of the hydrogel. The gel formation was verified if flow was not observed within 60 ​s after inverting the bottle.

### 2.14 *In vitro* degradation of hydrogels

The hydrogels were divided into two groups and weighed, followed with immersion in PBS solutions (0.01 ​M) of pH 7.4 and pH 6.5 containing 300 mU/mL hyaluronidase, respectively. At different time points after immersion, the samples were weighed after the superficial water of hydrogel was removed using a filter paper. The degradation rate was calculated using the following equation: Weight loss ratio (%) = (W_0_ -Wm)/Wm, where W_0_ and Wm represent the initial weight of hydrogel and the weight of hydrogel at different time points after immersion, respectively. The experiments were conducted in triplicate.

### 2.15 *In vitro* biological assessment of hydrogels

MC3T3-E1 pre-osteoblasts were co-cultured with hydrogels to perform biological assessment. Briefly, cell suspensions at a density of 1 ​× ​10^4^ ​cells per tube were added into hydrogels and mixed gently. The concentration of hydrogels in the mixture was 1 ​μg/mL. The mixtures of hydrogels and cell suspensions were plated in a culture dish before they begun to solidify. The cell-encapsulated gels were covered with cell culture medium and incubated at 37 ​°C. After 1, 7, and 14 days, the cells were stained with propidium iodide (red fluorescent dye) and calcein acetoxymethyl ester (green fluorescent dye) for CLSM observation, respectively. To test the cytotoxicity of hydrogel materials, the cells were incubated for 1, 3 or 7 days with different concentrations of hydrogel materials. The cell viability was measured using the MTT [3-(4,5-dimethylthiazol-2-yl)-2,5-diphenyltetrazolium] assay.

The MC3T3-E1cells were treated with PBS (PBS group), polyplexes targeting VEGF-A gene (V group), polyplexes targeting TGF-β1 gene (T group), and polyplexes targeting both VEGF-A and TGF-β1 genes (VT group). The cells transfected with different polyplexes were encapsulated into HA hydrogels. Briefly, cells transfected with different polyplexes for 48 ​h were harvested by trypsinization, centrifuged, and resuspended in 1 ​μg/mL hydrogel at 37 ​°C under aseptic conditions, resulting in a 20% (w/v) hydrogel containing 2 ​× ​10^6^ ​cells. The hydrogel containing no cells was used as control.

### 2.16 Calvarial defect model

All surgical experiments strictly abided by the guidelines of the Institutional Animal Care and Use Committee of Southern Medical University. A full-thickness (2.7 ​mm in diameter) defect was drilled on 8 ​– ​12-week-old C57BL/6 male mice on the parietal bone using a trephine drill (Changzhou, China) with care to avoid potential damage to the dura mater. The defect site was rinsed extensively with saline to remove bone fragments and implanted with the prepared hydrogels. The incisions were sutured, and all animals were subcutaneously administered buprenorphine at 0.1 ​mg/kg body weight once a day for 3 days. The animals drank water containing trimethoprim/sulfamethoxazole for 1 week to prevent postoperative infection. All of the mice were sacrificed after implantation for 4 or 8 weeks, and the skulls were removed and fixed in 4% paraformaldehyde.

### 2.17 MicroCT scanning and analysis

Bone defect specimens were collected and immersed in the formaldehyde solution (4%) for 1 ​d at 25 ​°C. Microcomputed tomography (μCT) analysis was performed for all the samples using a μCT 80 (Scanco Medical, AG, Switzerland) instrument with an isotropic voxel size of 36 ​μm (145 ​μA, 55 ​kVp). DICOM images of each sample were reconstructed using Mimics Research 17.0 (Materialise), Bone volume (mm^3^) and bone density (average Hounsfield Unit, HU) within a chosen disk-shaped volume of interest (VOI, 2.7 ​mm in diameter and 0.5 ​mm in height) representing the original defect, and the percentage of newly formed bone surface area was analyzed and compared to the original defect.

### 2.18 Histological evaluation

After μCT scanning, the parietal bones were decalcified and paraffin-embedded. Then, 5 ​μm longitudinal sections of bone samples were subjected to hematoxylin and eosin (H&E) staining, Masson trichrome staining, and immunohistochemical staining, respectively. The images were captured using bright-field microscopy (E800 microscope, Nikon, Japan). The deparaffinized sections were processed with citric acid for antigen retrieval, thereafter incubated with the primary antibody against osteocalcin (OCN) and CD31, and were detected by the HRP/DAB kit (DAKO, K5007). The sections were further counterstained with Mayer's hematoxylin.

### 2.19 Statistical analysis

For each sample, data was showed mean ​± ​standard deviation (SD) and was analyzed using Student's t-test for comparing means from two independent sample groups using SPSS (v. 10.0) software (∗*p* ​< ​0.05, ∗∗*p* ​< ​0.01, and ∗∗∗*p* ​< ​0.001).

## 3 Results

### 3.1 Preparation and characterization of polyplexes

**Fig. 2:**
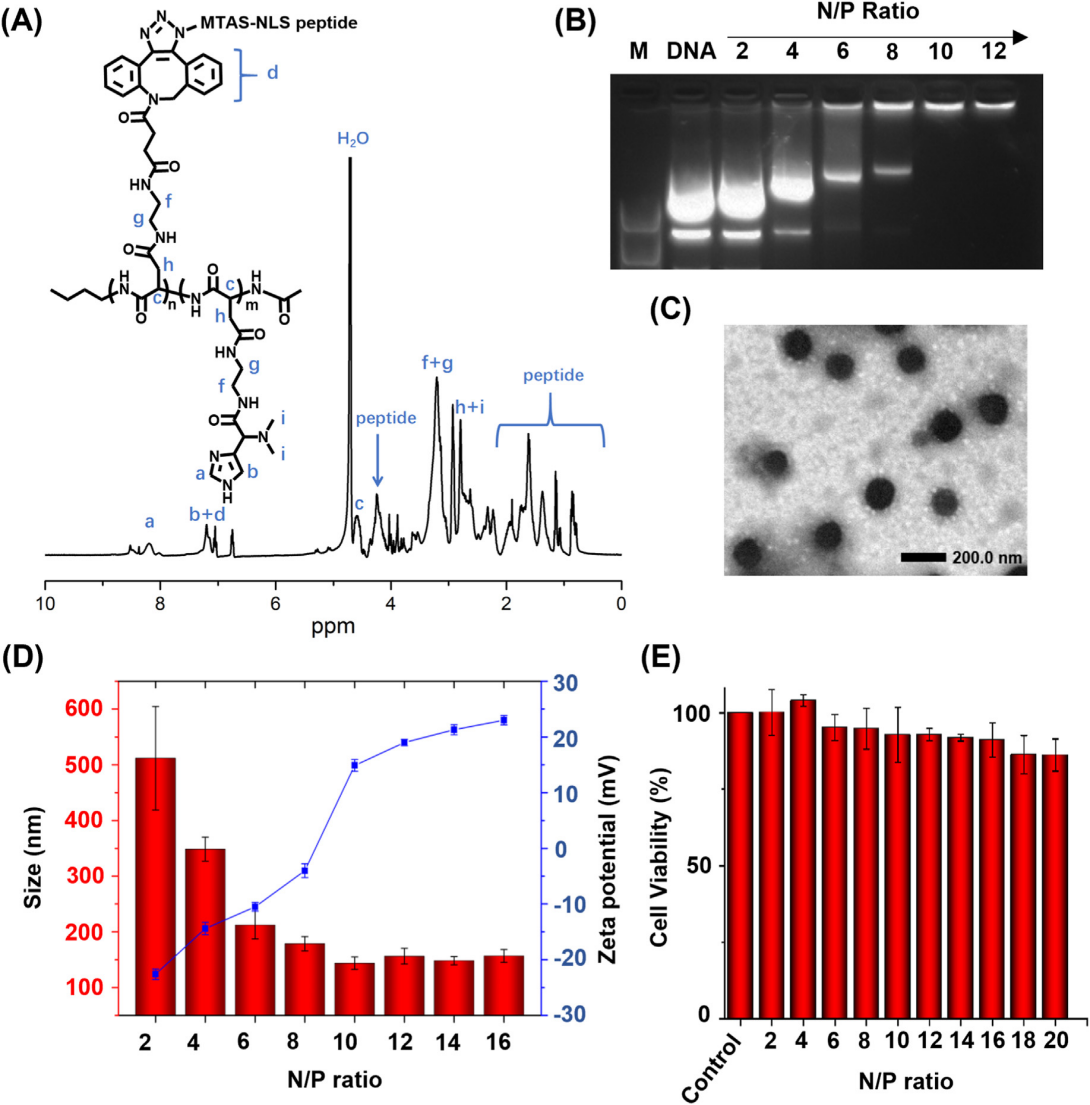
Preparation and characterization of copolymer and nanoparticle. (A) ^1^H NMR spectrum of copolymer PAsp(MTAS-NLS-*co*-DMH). (B) Electrophoretic mobility of plasmids in agarose gel after complexation with PND polymers. (C) Transmission electron microscopy (TEM) image of nanoparticles at a N/P ration of 10. Scale bars, 200 ​nm. (D) Sizes and zeta potentials of PND/pDNA-NPs at different N/P ratios. (E) Cytotoxicity of PND/pDNA-NPs at different N/P ratios to MC3T3-E1 cells. Mean SD (n ​= ​3). The PND polymer and pDNA indicate polymer PAsp(MTAS-NLS-*co*-DMH) and plasmid DNA, respectively.

The copolymer polyaspartate(N-(dibenzocyclooctyne)-*co*-(N′, N′- dimethyl-histidine), referred to here as PAsp(DBCO-DMH), was first synthesized and characterized (Figs. S1–S2). The appearance of characteristic peaks for histidine group (a, 7.8 ​ppm, -HNC***H*** = N-; b, 7.0 ​ppm, -HNC***H*** = C-) and DBCO group (d, 7.2 ​ppm, Ar) confirmed the successful synthesis of PAsp(DBCO-DMH). Then, the N_3_-modified peptide NLS-MTAS was conjugated to PAsp(DBCO-DMH) through azide-alkyne cycloaddition (Fig. 2A and Scheme S1). The new characteristic peaks at 0.8–2.0 ​ppm and 4.21 ​ppm assigned to NLS-MTAS peptide. These results suggest the successful synthesis of polymer PAsp(NLS-MTAS-*co*-DMH), referred to here as polymer PND. Next, the polymer PND was used to complex with CRISPRa plasmid DNA (CRISPRa pDNA) to form a polyplex (Fig. 1). The complexation capacity of polymer PND with pDNA at various N/P ratios was determined by using agarose gel retardation assay (Fig. 2B). The zeta potentials of PND/pDNA-NPs increased with the N/P ratio and showed a positive charge at N/P ratios above 10. The size of polyplexes decreased as the increase of N/P ratio and kept constant (∼135 ​nm) at N/P ratios above 10, showing a uniform and round morphology observed using transmission electronic microscopy (TEM) (Fig. 2C). At N/P ratios below 10, the pDNA bands with different migration distances were observed because of the different complexation states caused by incomplete complexation of pDNA. In contrast, the pDNA bands were completely retarded when the N/P ratio was greater than or equal to 10, which was due to the negative charges of pDNA being completely neutralized by cationic polymers PND. These results are in line with that of zeta potential (Fig. 2D). The polyplexes showed low cytotoxicity (>90%) even at a high N/P ratio of 16 (Fig. 2E). Although the cell viability of PND/pDNA-NPs at N/P ratios of 2 was greater than 100%, it showed no statistical significance compared with control group. The polyplexes at an N/P ratio of 10 had a smaller size of ∼143 ​nm and a lower zeta potential of +14.9 ​mV, which were in favor of cellular uptake. In addition, the polyplexes at an N/P ratio of 10 showed excellent colloidal stability in cell culture medium. Therefore, the polyplexes were prepared at an N/P ratio of 10 in subsequent experiments.

### 3.2 Cellular uptake of polyplexes

**Fig. 3:**
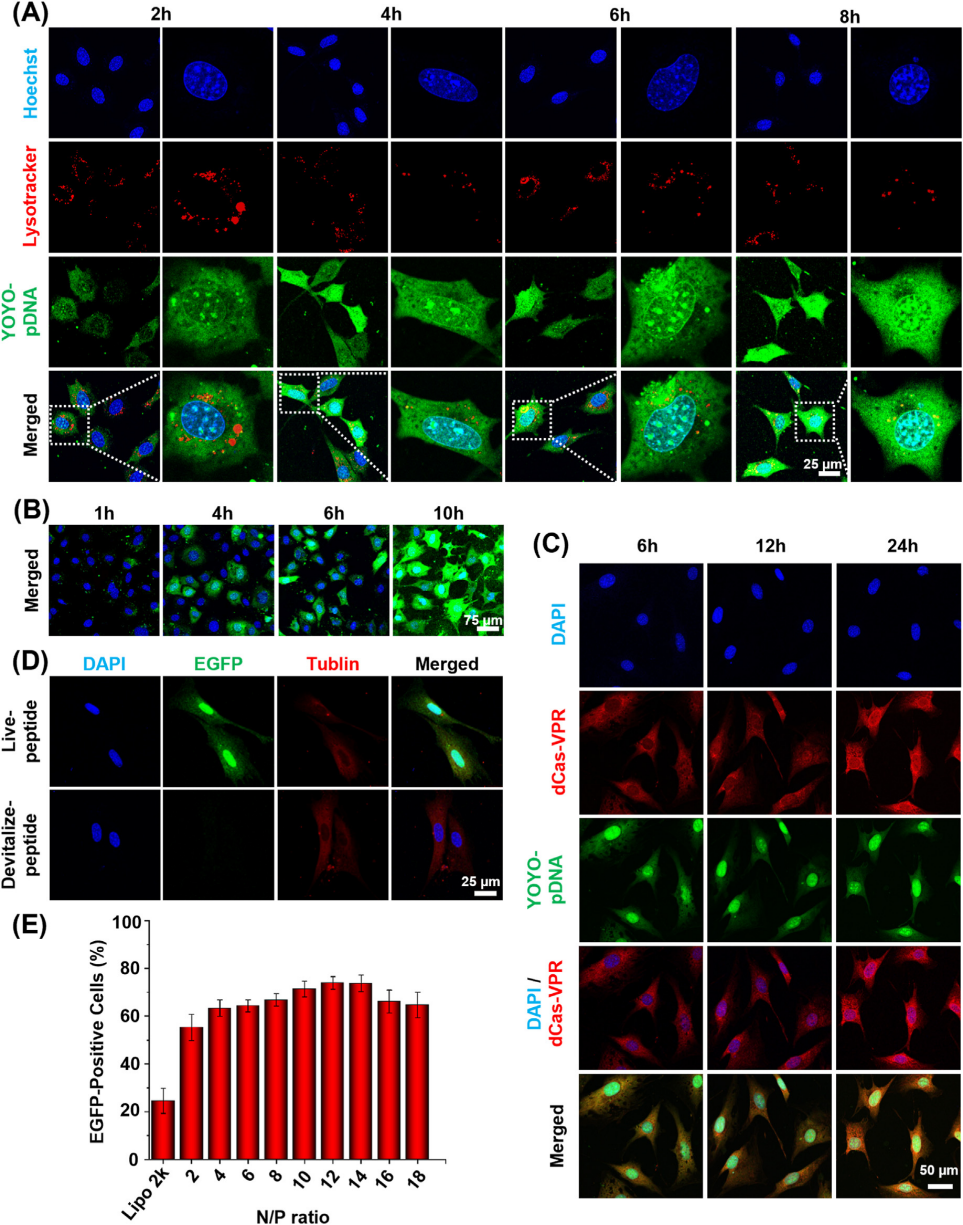
Mechanism and characterization of the intracellular trafficking of PND/pDNA-NPs. (A) Lysosomal escape of PND/pDNA-NPs. (B) Time-lapse CLSM images of intracellular trafficking of PND/pDNA-NPs. (C) Immunofluorescence staining of dCas9-VPR protein. (D) Confocal laser scanning microcopy (CLSM) of cells transfected with PND/pDNA-NPs modified with live peptide MTAS-NLS and devitalize peptide, respectively. (E) Flow cytometry analysis of EGFP-positive cells transfected with PND/pEGFP-NPs (mean ​± ​SD, n ​= ​3).

To track the intracellular fate of CRISPRa pDNA, the nuclei, lysosomes, and pDNAs were labeled with Hoechst 33342 (blue fluorescence), LysoTracker™ Deep Red (red fluorescence), and YoYo-1 dye (green fluorescence), respectively. To clarify the intracellular distribution of pDNA and dCas9 protein, we magnified the cell images and quantitatively analyzed the fluorescence signals of nucleus and pDNA. The non-overlapping region of green and red fluorescence indicated the lysosomal escape of pDNAs, and the increase of green fluorescence with time in the range of blue fluorescence indicated the accumulation of pDNA in the nuclei (Fig. 3A, B and S8A). The lysosomal escape of PND/pDNA-NPs is due to the pH-sensitive DMH moieties of PND polymer which rupture the lysosomes via a proton sponge effect [23d]. At 24 ​h after transfection, the pDNAs and Cas9 proteins mainly located in the nuclei (Fig. 3C, S8B), which is closely related to the nuclear-trafficking peptide NLS-MTAS [24a]. The constant green fluorescence of pDNA was due to the reason that the no pDNA were internalized into cells after cell culture medium containing PND/pDNA-NPs was replaced with fresh medium without nanoparticles. To verify the role of nucleus localizing peptide NLS-MTAS, a PND polymer modified with scrambled NLS-MTAS sequence instead of the active one was used to deliver pDNA encoding enhanced green fluorescence protein (EGFP). The tubulins were stained red to display cell morphology.

The polymer PND with unscrambled NLS-MTAS peptide resulted in significant EGFP production. In contrast, no obvious EGFP gene expression was observed in the cells treated with polymer with the scrambled peptide (Fig. 3D). To quantify the delivery efficiency of polymer PND, the cells transfected with PND/pEGFP-NPs were subjected to flow cytometry analysis. The percentage of EGFP-positive cells increased with the N/P ratio and reached a plateau of ∼75% at N/P ratios between 8–12. By contrast, the commercial transfection agent Lipofectamine only resulted in 24.5% of EGFP-positive cells. These results suggest that the polymer PND can effectively deliver pDNA into the nuclei to produce target proteins, indicating that pH-sensitive DMH and nucleus localizing peptide NLS-MTAS are vital for lysosomal escape and nuclear translocation of pDNA, which are favorable for highly efficient dCas9/sgRNA-based gene activation. The polyplexes with an N/P ratio above 10 showed no significant increases in the transfection efficiency (Fig. 3E). Therefore, the PND/pDNA-NPs prepared at an N/P ratio of 10 were used in following studies based on comparative analysis of the delivery efficiency and cytotoxicity of PND/pEGFP-NPs at various N/P ratios.

### 3.3 TGF-β1 and VEGF-A specific gene activation

The CRISPR-dCas9 system can specifically activate target gene expression, guiding by sgRNA complementary to the upstream sequence (promotor region) [13]. We designed and synthesized 19 strands of psgRNA targeting mouse *TGF-β1* (T-sgRNA) and *VEGF-A* (V-sgRNA) genes, listed in Table S1. The *TGF-β1* and *VEGF-A* genes were targeted by 9 and 10 strands of sgRNAs, respectively. The highest levels of TGF-β1 and VEGF-A mRNAs were recorded in the cells treated with T8-sgRNA and V9-sgRNA, increasing mRNA levels by 4.88 and 4.82 folds (Fig. S4A) in comparison to the control group, respectively. The combined treatment of several sgRNAs targeting TGF-β1 gene showed lower mRNA level than the T8-sgRNA alone treatment. Similarly, the combined treatment of several sgRNAs targeting VEGF-A showed no significant increase in mRNA level of VEGF-A compared to V9-sgRNA alone treatment (Fig. S4B). To optimize the use of T8-sgRNA and V9-sgRNA, their dosage effect was evaluated. The highest level of TGF-β1 mRNA was observed in the cells receiving 2.5 ​μg of T8-sgRNA(Fig. S4C). Although the VEGF-A mRNA levels increased with the amount of V9-sgRNA, the PND/pDNA-NPs showed cytotoxicity to cells at the amounts of above 4.5 ​μg. The combined treatment of T8-sgRNA and V9-sgRNA did not enhance TGF-β1 mRNA level but significantly increased VEGF-A mRNA level by 6.5 folds compared to single treatments (T8-sgRNA and V9-sgRNA treatments). Notably, the increase of V9-sgRNA amount in the combined treatment of T8-sgRNA and V9-sgRNA reduced the TGF-β1 and VEGF-A mRNA levels (Fig. S4D). Therefore, the T8-sgRNA and V9-sgRNA were combined at doses of 2.5 ​μg and 1 ​μg, respectively. In subsequent studies, the T-sgRNA and V-sgRNA indicated T8-sgRNA and V9-sgRNA, and their combination was named TV-sgRNA. Thus, the polyplexes formed with polymers PND and pDNAs encoding CRISPRa-dCas9 and TV-sgRNA were named PND/TV-NPs. Similarly, the polyplexes carrying pDNA encoding T-sgRNA or V-sgRNA were named PND/T-NPs or PND/V-NPs, respectively.

**Fig. 4:**
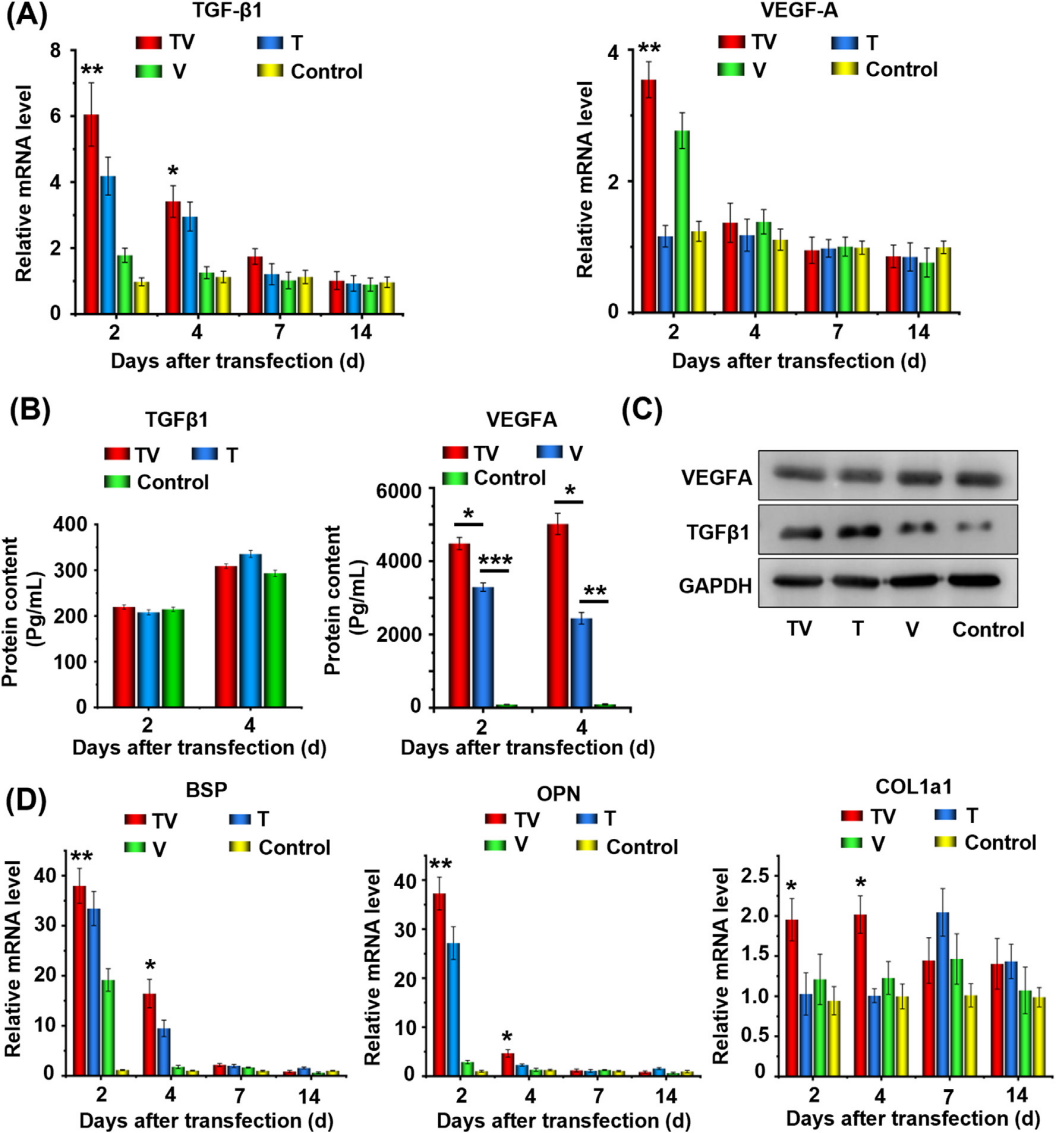
Activation profiles of VEGF-A, TGF-β1 and osteogenic proteins in the cells transfected with PND/pDNA-NPs targeting VEGF-A and TGF-β1. (A) Relative mRNA levels of VEGF-A and TGF-β1 genes at different time points after transfection. (B) Extracellular contents of VEGF-A and TGF-β1 proteins determined by ELASIA assay at different time points after transfection. (C) Intracellular contents of VEGF-A and TGF-β1 proteins determined by Western-blot assay at 2 ​d after transfection. (D) Relative mRNA levels of BSP, OPN and COL1a1 at different time points after transfection. The relative mRNA levels are determined by normalizing to those of the control cells without PND/pDNA-NPs transfection. The data represent mean ​± ​SD of three independent culture experiments. The T, V, and TV indicate the PND/T-NPs, PND/V-NPs, and PND/TV-NPs treatments, respectively. ∗*p* ​< ​0.05, ∗∗*p* ​< ​0.01 versus control group.

Next, the time-dependent effect of PND/TV-NPs treatment on the TGF-β1 and VEGF-A gene expressions was investigated (Fig. 4A and B). In comparison to control treatment, the single treatment with PND/T-NPs increased the TGF-β1 mRNA levels to 4.2 folds at 2 ​d after transfection but did not induce VEGF-A gene expression, and the single treatment with PND/V-NPs increased the TGF-β1 mRNA levels to 1.8 folds and VEGF-A mRNA level to 2.8 folds, respectively. Notably, the combined treatment with PND/TV-NPs increased the TGF-β1 mRNA levels to 6.0 folds and VEGF-A mRNA level to 3.5 folds. The inductive effect of the CRISPRa system on TGF-β1 and VEGF-A mRNA levels disappeared at 7 ​d and 14 ​d after transfection, respectively. The extracellular and intracellular proteins of TGF-β1 and VEGF-A were detected by ELISA analysis (Fig. 4B) and Western blot (Fig. 4C). All CRISPRa treatments increased the intracellular content of TGF-β1 protein, but no significant difference was observed between PND/T-NPs and PND/TV-NPs treatments. However, the intracellular content of VEGF-A protein was not influenced by each CRISPRa treatment. As for their extracellular contents, the PND/T-NPs and PND/TV-NPs treatments showed no effect on the TGF-β1 contents, but the PND/V-NPs and PND/TV-NPs treatments increased the contents of VEGF-A by 3416 folds and 4311 folds compared to control treatment at 4 ​d after transfection, showing significant synergism on VEGF induction. Thus, the single treatment PND/T-NPs and PND/V-NPs can effectively induce *TGF-β1* and *VEGF-A* gene expressions, respectively, and the combined treatment with PND/TV-NPs strengthens this effect.

Next, we investigated whether the activation of TGF-β1 and VEGF-A can enhance osteogenesis of pre-osteoblasts. The activations of bone sialoprotein (BSP), osteopontin (OPN), and collagen type I alpha1 (COL1a1) reflect the osteogenic capacity of implanted cells [35]. Compared to single treatments of PND/T-NPs and PND/V-NPs, the combined sgRNA treatment of PND/TV-NPs increased the mRNA levels of BSP to 1.7 folds and 8.0 folds at 4 ​d after transfection, OPN to 1.8 folds and 15 folds at 2 ​d after transfection (Fig. 4D), respectively, showing additive or synergistic effects on the osteogenesis. The inductive effect on BSP and OPN of these nanoparticles almost disappeared 7–14 ​d after transfection. Notably, the single sgRNA treatments hardly increased the COL1a1 gene expression 2–4 ​d after transfection, but the combined treatment significantly increased its expression to ∼2.0 fold compared to the control treatment. The greatest levels of COL1a1 mRNA appeared at 7 ​d after transfection in the cells receiving with single sgRNA treatments, which was later than that in the cells receiving combined sgRNA treatment. These results demonstrate that the combined treatment with PND/TV-NPs synergistically enhances the expression levels of osteogenesis-related genes.

### 3.4 *In vitro* osteogenic potential

**Fig. 5:**
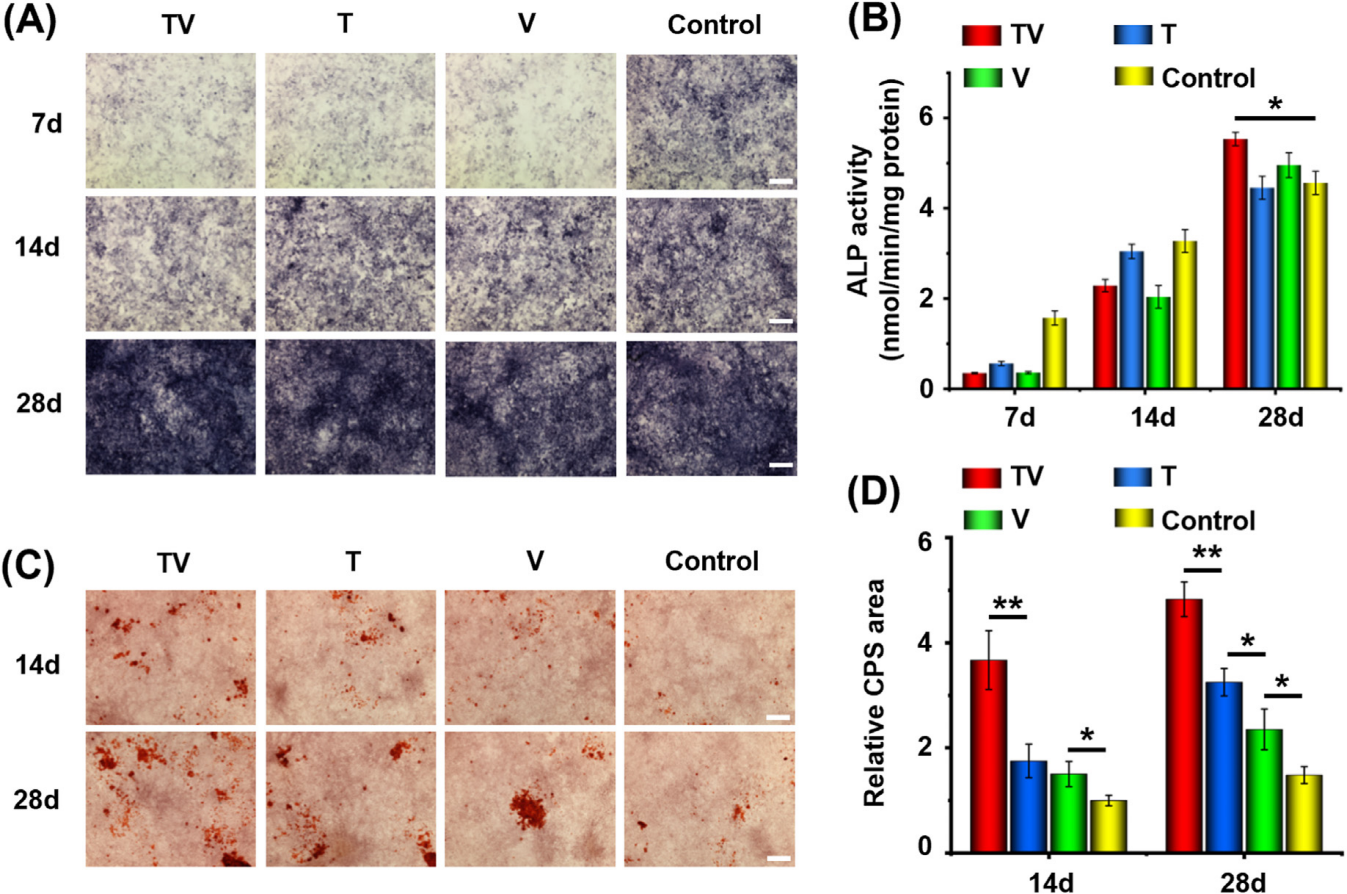
Osteogenic potential of MC3T3-E1 cells transfected with non-viral CRISPRa system targeting VEGF-A and TGF-β1. (A) Alkaline phosphatase (ALP) staining and (B) Activity analysis of ALP in the cells receiving different treatments at 14 and 28 ​d after transfection. (C) Deposition of calcium phosphate salts (CPS) determined through Alizarin red staining and (D) Contents of CPS in the panel C in the cells receiving different treatments at 14 and 28 ​d after transfection. Cells were cultured in the osteoinduction medium to induce osteogenesis. The data are mean ​± ​SD of three independent culture experiments, ∗*p* ​< ​0.05, ∗∗*p* ​< ​0.01. The T, V, and TV indicate the PND/T-NPs, PND/V-NPs, and PND/TV-NPs treatments, respectively. Scale bars: 50 ​μm.

The osteoblastic activity and mineralization, which are indicated with the activity of alkaline phosphatase (ALP) and deposition of calcium phosphate salts (CPS), respectively, are important indicators to directly evaluate osteogenic potential of pre-osteoblasts and MSCs [36]. All treatments suppressed the ALP activities 7–14 ​d after transfection, whereas the combined treatment showed the greatest ALP activity at 28 ​d after transfection (Fig. 5A and B). These results indicated that the ALP activity was inhibited at the early stage after transfection but was enhanced in the late stage by the up-regulation of TGF-β1 and VEGF-A, which agrees with a previous work [37]. The cell layer in the combined treatment (PND/TV-NPs) showed greater CPS area than that in any single treatment (PND/T-NPs and PND/V-NPs) at 28 ​d after transfection (Fig. 5C and D), with a CPS area increase of 3.83 folds, 2.25 folds, and 1.35 folds when compared to control group, respectively. Thus, the increases of ALP activity and CPS content in the cells treated with PND/TV-NPs were closely related to upregulation of TGF-β1 and VEGF-A.

### 3.5 Preparation and characterization of dual crosslinked hydrogels

**Fig. 6:**
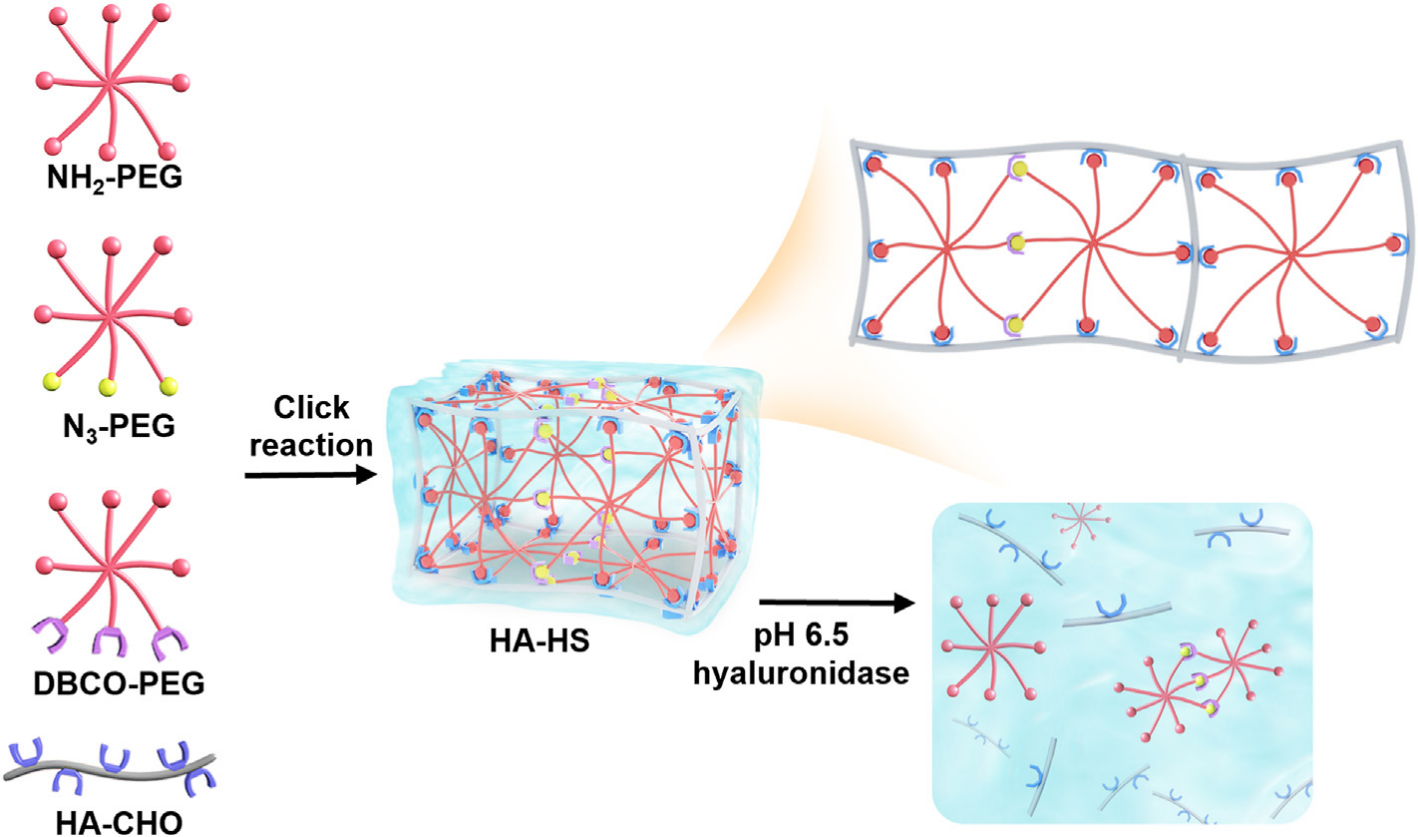
Schematic of preparation and degradation of crosslinked hydrogels HA-HS. The aldehyde derivative of HA (HA-CHO) is mixed with NH_2_-PEG, N_3_-PEG, and DBCO-PEG to prepare dual-crosslinked hydrogel HA-HS through the formations of hydrozone and strain-promoted azide-alkyne cycloadditions (SPAAC) structures. The NH_2_-PEG, N_3_-PEG, and DBCO-PEG indicate the oxyamine-terminated 8-arms PEG, oxyamine- and N_3_-terminated 8-arms PEG, and oxyamine- and DBCO-terminated 8-arms PEG, respectively. The disassembly of hydrogel occurs in the presence of low pH (pH 6.5) and hyaluronidase through the breakage of pH-sensitive hydrozone bond and the hyaluronidase-triggered HA degradation.

**Fig. 7:**
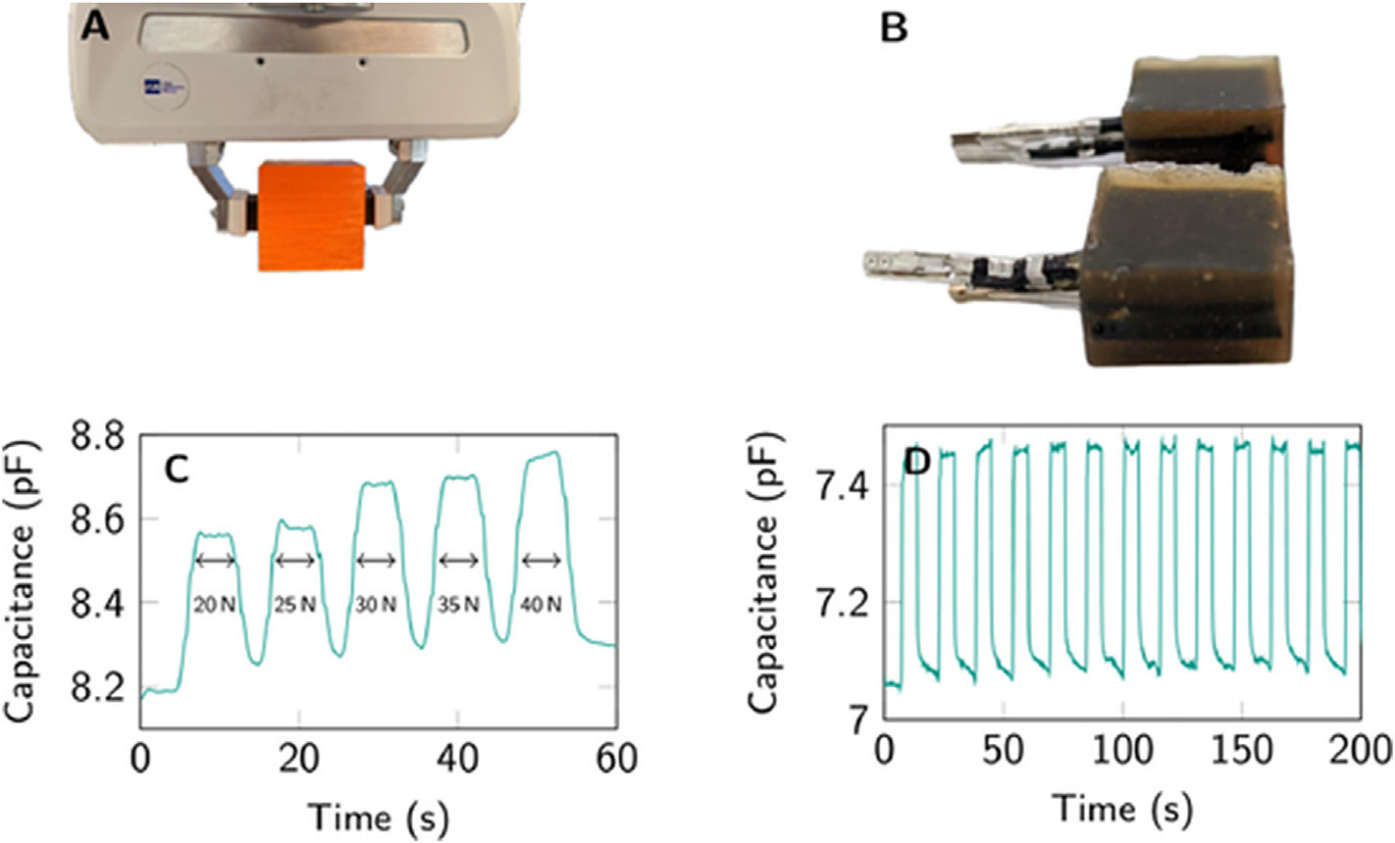
Characterizations of different hydrogels. (A) FT-IR spectra of HA- HS, HA-DBCO and HA-N_3_ hydrogels. (B) Gelation times of hydrogels at different mass ratio of HA-CHO to NH_2_-PEG. (C) Gelation times of hydrogels at different mass ratio of HA-CHO to NH_2_-PEG to N_3_-PEG to DBCO-PEG. (D) Weight loss ratios of hydrogels in the presence of 300mU/mL of hyaluronidase under pH 7.4 and pH 6.5, respectively. (E) Scanning electron microcopy (SEM) images of hydrogels. (F) Three-dimensional images of MC3T3-E1 cells in the hydrogels HA–HS–0.25. The live and dead cells were stained green and red, respectively. The HA-CN indicates single-crosslinked hydrogel which was prepared at a mass ration of HA-CHO to NH_2_-PEG of 1 : 1.5. The HA–HS–0.1 indicates dual-crosslinked hydrogel which was prepared at a mass ration of HA-CHO to NH_2_-PEG to N_3_-PEG to DBCO-PEG of 1 : 1.5: 0.1 : 0.1. The HA–HS–0.25 indicates dual-crosslinked hydrogel which was prepared at a mass ration of HA-CHO to NH_2_-PEG to N_3_-PEG to DBCO-PEG of 1 : 1.5: 0.25 : 0.25.

To encapsulate and implant the engineered pre-osteoblasts into the bone defect site, the HA-based hydrogel with a dual crosslinking structure was prepared and characterized (Fig. 6, Fig. 7, and S5-7). We first synthesized an aldehyde derivative of HA (HA-CHO) by oxidizing the vicinal hydroxyl groups in the HA to dialdehyde in the presence of sodium periodate. The characteristic resonance peaks at 3.6–3.8 ​ppm for -C***H***, 3.4–3.6 ​ppm for -C***H***_*2*_, and 7.8–7.9 ​ppm for aldehyde group indicated the successful synthesis of the HA-CHO (Fig. S5A). The conversion efficiency of the vicinal hydroxyl to aldehyde was 49.1% according to the integral value of characteristic peaks. Next, we synthesized the azide-modified PEG (N_3_-PEG) and cyclooctyne-modified PEG (DBCO-PEG) through the amidation reaction by which the NH_2_ in the 8-arm oxyamine-PEG (NH_2_-PEG) reacted with *p*-azidobenzoic acid and dibenzocyclooctyne-succinimide, respectively. In the ^1^H NMR spectrum, the characteristic resonance peaks at 3.4–3.6 ​ppm for PEG (a, b, -C*H*_2_), at 7.8–7.9 ​ppm for benzene ring in DBCO (d, e, -C*H*), and at 7.75–7.85 ​ppm for benzene ring in *p*-azidobenzoic acid (c, d, -C*H*) confirmed the successful synthesis of N_3_-PEG and DBCO-PEG (Figs. S5B and C). According to the integral value of characteristic peaks, 33.5% and 37.9% of amino groups in the NH_2_-PEG were converted to DBCO and N_3_, respectively. Finally, these polymers were used to prepare a dual-crosslinked HA hydrogel (Fig. 6). One crosslinking structure was the pH-sensitive hydrozone bond between the aldehyde groups in the HA-CHO and oxyamine groups in the PEG polymers (e.g., NH_2_-PEG, N_3_-PEG, and DBCO-PEG); the other one was result of the strain-promoted azide-alkyne cycloadditions (SPAAC) between N_3_-PEG and DBCO-PEG. The slow-reacting aldehyde-oxyamine conjugation and fast-reacting N_3_-DBCO conjugation were combined to produce a dual-crosslinked hydrogel with tunable mechanical and biochemical properties, which are favorable for cell encapsulation and proliferation [38]. Moreover, the combination of stable SPAAC and pH-labile hydrozone linkages allowed unique degradation control to encourage cell expansion in the hydrogel matrix [38].

Thus, the HA-CHO was mixed with oxyamine-terminated polymers NH_2_-PEG and N_3_-PEG in sequence at room temperature to prepare the single-crosslinked hydrogel HA-N_3_ through the formation of hydrozone bonds between oxyamine and aldehyde groups. Similarly, the single-crosslinked hydrogel HA-DBCO was prepared using HA-CHO, NH_2_-PEG and DBCO-PEG. Afterward, the HA-N_3_ and HA-DBCO were mixed to prepare the dual-crosslinked hydrogel HA-Hydrozone-SPAAC, referred to here as HA-HS. HA-HS hydrogels were characterized by Fourier Transform Infrared Spectroscopy (FTIR) analysis. As shown in Fig. 7A, compared to single-crosslinked hydrogels HA-N_3_ and HA-DBCO, the disappearance of the characteristic peak at 2100-2200 ​cm^−1^, which is ascribed to azido and alkynyl groups of DBCO, indicated successful click reactions between DBCO and N_3_.

To optimize the formation of HA-HS, we comparatively analyzed the morphologies and physicochemical properties of hydrogels prepared at different mass ratios of HA-CHO to crosslinking agents (e.g., NH_2_-PEG, N_3_-PEG, and DBCO-PEG). In the presence of NH_2_-PEG, the gelation time shortened with the mass ratio of HA-CHO to NH_2_-PEG (M_HA-CHO_: M_NH2-PEG_) (Fig. 7B). The hydrogel prepared at the M_HA-CHO_: M_NH2-PEG_ of 1 : 1.5, referred to here as HA-CN showed a proper gelation time (25 ​min), allowing the introduction of the other crosslinking structure based on SPAAC linkage. After HA-CHO and NH_2_-PEG (M_HA-CHO_: M_NH2-PEG_ ​= ​1 : 1.5) were mixed, the N_3_-PEG and DBCO-PEG were added at different mass ratios (M_HA-CHO_: M_NH2-PEG_: M_N3-PEG_/_DBCO-PEG_) to obtain HA-N_3_ and HA-DBCO solution, respectively. Finally, the HA-N_3_ and HA-DBCO were mixed at 37 ​°C to prepare dual-crosslinked hydrogels at different mass ratios of HA-CHO hydrogel to N_3_-PEG and DBCO-PEG (M_HA-CHO_: M_NH2-PEG_: M_N3-PEG_: M_DBCO-PEG_). The dual-crosslinked hydrogels at M_HA-CHO_: M_NH2-PEG_: M_N3-PEG_: M_DBCO-PEG_ of 1 : 1.5: 0.1 : 0.1 and 1 : 1.5: 0.25 : 0.25 were named HA–HS–0.1 and HA–HS–0.25, respectively, showing gelation times of 16 ​min and 8 ​min, respectively (Fig. 7C). The equilibrium swelling ratios of HA-CN, HA–HS–0.1, and HA–HS–0.25 were ∼57%, ∼46%, ∼36% (Fig. S6), respectively. The swelling ratio was closely associated with the rigidity of hydrogel. Furthermore, the rheological tests demonstrated that the storage modulus (*G′*) is much higher than loss modulus (*G″*) for all hydrogels. The *G′* values of HA-CN, HA–HS–0.1, and HA–HS–0.25 were 1700 ​Pa, 3400 ​Pa and 5500 ​Pa (Fig. S7), respectively, suggesting that the increase of fraction of N_3_ and DBCO can enhance the rigidity of hydrogel.

Biodegradation of hydrogels is mainly attributed to the degradation of polymeric backbone and cleavage of crosslinking bonds, which are influenced by the enzymes and local pH [39]. The biodegradable HA backbone and low pH-labile hydrozone bonds endow the dual-crosslinked hydrogels with biodegradability. Moreover, the combination of the cleavable crosslinker hydrozone bond and nondegradable crosslinker SPAAC assigned tunable degradation and stiffness properties to hydrogel, which favored the balance of promoting cell expansion and keeping 3D structure of hydrogel [40]. The HA-CN, HA–HS–0.1 and HA–HS–0.25 showed a largely linear weight loss in the presence of hyaluronidase, and lower pH conditions (pH 6.5) accelerated their degradation (Fig. 7D). The single-crosslinked hydrogels exhibited ∼30% and ∼60% weight loss at 27 ​d and 54 ​d after hyaluronidase treatment at pH 7.4. At lower pH, degradation increased, resulting in ∼50% and ∼96% of weight loss. In contrast, the addition of the non-degradable SPAAC crosslinker in the dual-crosslinked hydrogel HA–HS–0.25 reduced the weight loss to ∼25% after 27 ​d and ∼40% after 54 ​d in the presence of hyaluronidase treatment at pH 6.5. The morphology of crosslinked hydrogels was recorded using scanning electron microscopy (SEM). The single-crosslinked hydrogels HA-CN showed an interconnected porous structure with a pore size from several micrometers to ∼50 ​μm (Fig. 7E), resulting from the sublimation of water from the high-water-content hydrogel. Comparatively, the dual-crosslinked hydrogel HA-HS (e.g., HA–HS–0.1 and HA–HS–0.25) had a similar porous structure but thinner pore wall. These results indicate the combination of nondegradable structure (crosslinker SPACC) and degradable structures (HA backbone and low pH-labile hydrozone bonds) can control hydrogel degradation.

### 3.6 Cytocompatibility of dual crosslinked hydrogels

To investigate the cytocompatibility of different hydrogel with/without nanoparticles to MC3T3-E1 cells, the live/dead cell staining and cell counting kit-8 (CCK8) methods were performed (Fig. S9). Although the HA–HS–0.25 resulted in the greatest amounts of live cells, there were no significant difference in percentages of live cells among different hydrogels (e.g., HA-CN, HA–HS–0.1, and HA–HS–0.25). However, the cell proliferation in the HA–HS–0.25 hydrogel was greater than that in the HA–HS–0.1 and HA-CN hydrogels at 7 ​d and 14 ​d after implantation. The cells transfected with PND/pDNA-NPs showed similar behaviors (i.e., cytocompatibility and cell proliferation) with that without transfection. Moreover, we evaluated the cell behaviors in the different hydrogel with/without PND/TV-NPs (Fig. S10). In the absence of PND/TV-NPs, different hydrogels had the similar effect on the ALP activity, but the hydrogel HA–HS–0.25 resulted in the greatest CPS content. The transfection with PND/TV-NPs suppressed the ALP activities but enhanced the CPS contents, and the greatest CPS content was observed in the PND/TV-NPs-engineered cells encapsulated in the hydrogel HA–HS–0.25 incorporating PND/TV-NPs, which were consistent with the results of PND/TV-NPs-transfected cells without hydrogel encapsulation. After transfection with PND/TV-NPs, the cells encapsulated in the HA–HS–0.25 hydrogels showed the greatest mRNA levels of TGF-β1 and VEGF-A compared to that encapsulated in the HA-CN and HA–HS–0.1 hydrogels. Therefore, HA–HS–0.25 was used for the subsequent experiments due to its suitable and degradable porous structure, and excellent cytocompatibility. Furthermore, the cells almost covered the entire surfaces of the hydrogels and spread with numerous filopodia and cytoplasmic extensions, demonstrating that HA–HS–0.25 are favorable for cell adhesion and spreading in 3D culture (Fig. 7F). The hydrogel HA–HS–0.25 effectively encapsulated cells and showed excellent cytocompatibility to cells even at a high concentration of 10 ​μg/mL (Fig. S11), resulting in significant cell proliferation.

### 3.7 *In vivo* synergistic effect on osteogenesis

The pre-osteoblast MC3T3-E1 cells transfected with PND/TV-NPs were encapsulated and implanted into bone defects to promote osteogenesis through enhancing the expressions of TGF-β1 and VEGF-A. The mice were randomly divided into four groups, namely control (hydrogel loaded without cells), TGF-β1 (hydrogel loaded with PND/T-NPs transfected cells), VEGF-A (hydrogel loaded with PND/V-NPs transfected cells), and combination (hydrogel loaded with PND/TV-NPs transfected cells). The new bone areas were recorded and quantified by referring to the original defect size (2.7 ​mm in diameter) according to μCT images.

**Fig. 8:**
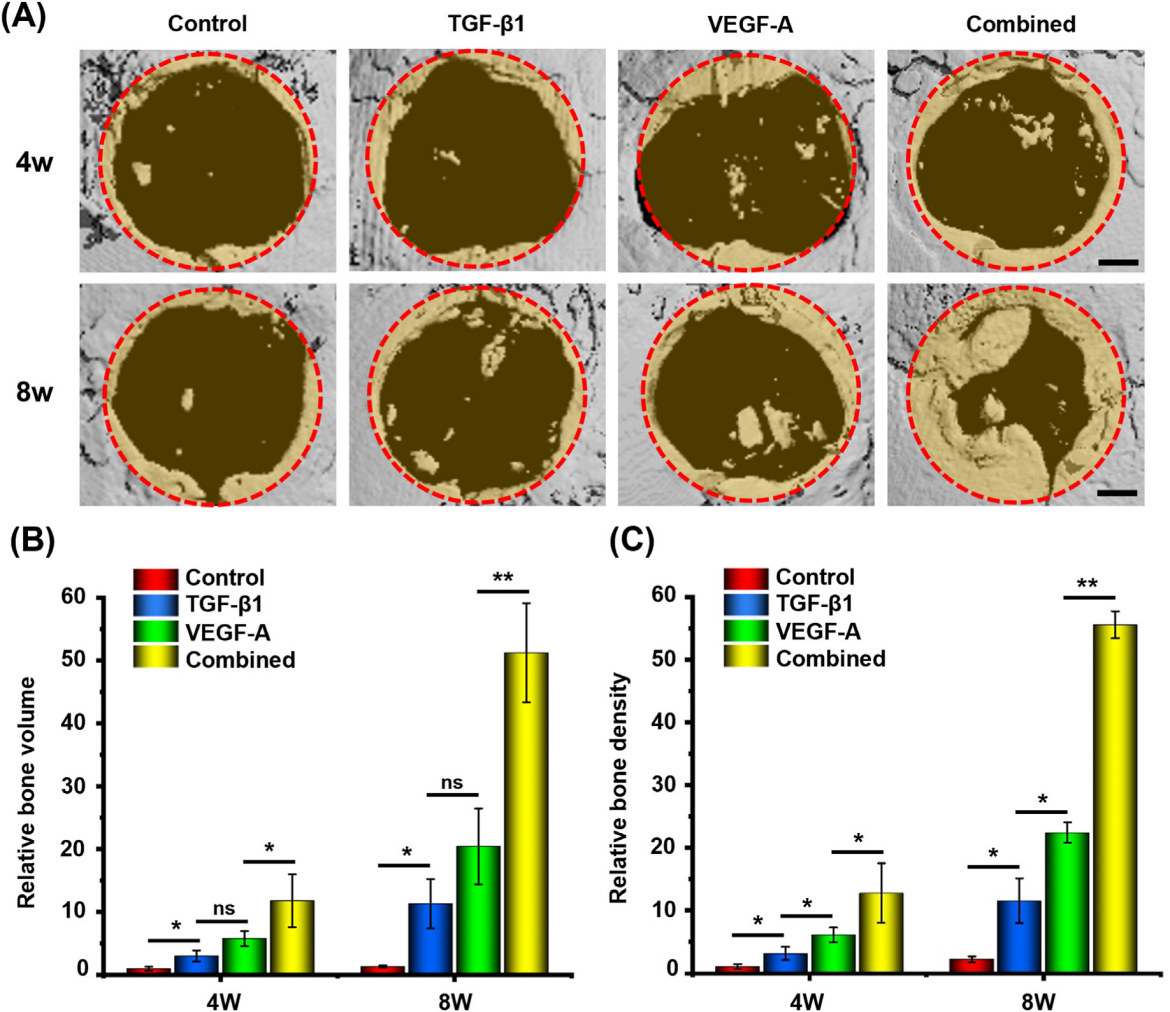
Bone healing capacity of non-viral CRISPRa system-engineered cells implanted with dual-crosslinked hydrogel. (A) Calvarial bone healing capacity evaluated by microcomputed tomography (Micro-CT) imaging at 4 and 8 weeks after implantation. (B) Quantitative analyses of the bone volumes and (C) Bone density of new bone formation at the bone defect site. After being transfected with PND/V-NPs, PND/T-NPs, and PND/TV-NPs, respectively, the MC3T3-E1 cells were implanted into bone defect site using dual-crosslinked hydrogel HA–HS–0.25. (∗∗*p* ​< ​0.01,∗*p* ​< ​0.05). The red dotted circles indicate the original edges of bone defects. Scale bars: 0.5 ​mm.

The TGF-β1 and VEGF-A treatments (single-activation of TGF-β1 and VEGF-A genes) increased the new bone volume by 11.3 folds and 21.4 folds 8 weeks after implantation compared to that of the control group (Fig. 8A and B). The mice in the combination group (combined activation of TGF-β1 and VEGF-A genes) showed the greatest new bone volume at all examined time points and increased the osteogenesis by 4.5 folds and 2.5 folds compared to that in the TGF-β1 and VEGF-A groups, respectively. The new bone densities in different mice presented the similar profiles (Fig. 8C). These results demonstrate that the combined activation of TGF-β1 and VEGF-A through nonviral CRISPRa systems can remarkably improve osteogenic capacity of pre-osteoblasts encapsulated in the dual-crosslinked hydrogels.

**Fig. 9:**
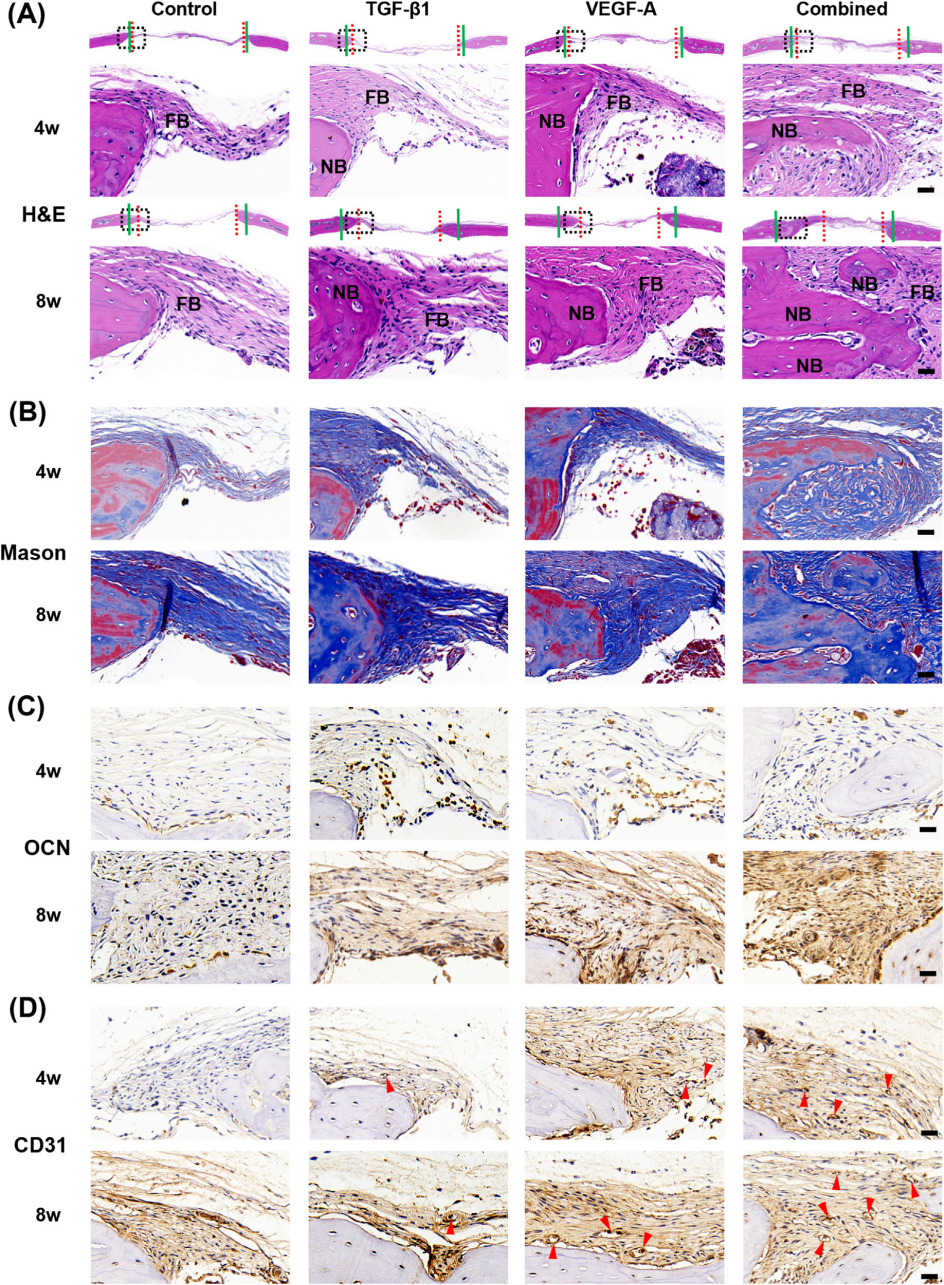
Histological and Immunohistochemical analysis. (A) Hematoxylin and eosin (H&E) staining. (B) Masson trichrome staining. (C–D) Immunohistochemical staining of osteocalcin (OCN) and CD31. The defects specimens were harvested at 4 and 8 weeks after implantation, followed with different staining analyses. The green solid and red dotted lines indicate that the fronts of original bone defect and new bone, respectively. The areas merked with dotted black rectangles were magnified in the H&E staining, Masson trichrome staining, and Immunohistochemical staining analyses. The nuclei and cytoplasm were stained blue and red in H&E staining, respectively. The calcified tissue and collagen were stained blue and red in Masson trichrome staining, respectively. The nuclei and target proteins (i.e., OCN and CD31) were stained blue and brown, respectively. Triangles indicate blood vessel-like structures. FB, fibroblast. NB, nascent bone. Scale bars: 25 ​μm.

Next, we performed the H&E and Masson trichrome staining to confirm the generation of new bone and osteoid. The mice in the TGF-β1 and VEGF-A groups showed greater contents of new bone and osteoid matrixes than those in the control group, but the greatest generation of new bone and osteoid matrixes was evidenced in the combination group (Fig. 9A and B). We quantified the relative gaps of defects by calculating the distance between the front edges of new bones. The mice in the combination group showed the narrowest gap at all examined time points and a 52% decrease in the distance of gap. However, the mice in the TGF-β1 and VEGF-A groups only reduced the distance of gap by 15%. The contents of OCN and CD31 indicate the capacity of bone metabolism and angiogenesis. The sections from mice in the TGF-β1 and VEGF-A groups showed moderate contents of OCN and CD31, while the greatest levels of OCN were recorded in that from the combination group (Fig. 9C). The greatest level of CD31 was observed in the combination group, which resulted in the most blood vessel-like structures marked with red arrowhead. Compared to control group, the single treatments (i.e., TGF-β1 or VEGF-A alone treatment) resulted in more blood vessel-like structures at 8 weeks after implantation, although they could not significantly enhance CD31 expression.

## 4 Discussion

The CRISPR-dCas9-based gene activation (CRISPRa) strategy is a powerful approach to specifically upregulate target gene expression. Although several studies have discussed its application in bone regeneration using viral vector-based CRISPRa systems, the non-viral vector-based CRISPRa system has so far not been exploited in enhancing bone formation. We developed a nonviral vector PND, composed of MTAS-NLS-grafted and dimethyl-histidine-grafted polyaspartate blocks, to deliver plasmids encoding CRISPR-dCas9 and sgRNA into pre-osteoblasts. The PND showed higher transfection efficiency than the commonly used commercial reagent Lipofectamine, which may be due to following reasons: i) the hydrophobicity of partly deprotonated amino groups in the methylated histidine residues under physiological conditions that facilitate integration of polyplexes and adherence to cell membranes; ii) the proton sponge effect of methylated histidine residues in the acidic lysosome that allows lysosomal escape with subsequent release of pDNA into the cytoplasm [23d]; iii) the nucleus translocation of fusion peptide MTAS-NLS a), [41]. The significant expression of dCas9 was confirmed in the cells treated with PND/pDNA-NPs polyplexes encoding dCas9 protein. Thus, the polymer PND provides a useful nanoplatform to activate target gene expression through CRISPRa mechanism.

Consequently, the TGF-β1 and VEGF-A expression levels are substantially increased by non-viral CRISPRa system (PND/TV-NPs) targeting TGF-β1 and VEGF-A genes, respectively. The activation of VEGF-A significantly increases TGF-β1, consistent with previously reported studies [42]. It was noted that exogenous TGF-β1 increased the VEGF-A mRNA level in the previously reported study [43]. However, in this study the increase of TGF-β1 hardly influences the VEGF-A mRNA level, possibly because the action mode of endogenous form of TGF-β1 is different from that of the exogenous form [44]. The CRISPRa treatments have no effects on intracellular contents of VEGF-A protein but result in significant increases of its extracellular content, which may be due to the reason that VEGF-A proteins are secreted out of cells to act on their receptors. Notably, the combined activation of TGF-β1 and VEGF-A results in greater levels of mRNA and extracellular protein of VEGF-A than single activation of either VEGF-A or TGF-β1 alone. These results demonstrate that the combined activation of TGF-β1 and VEGF-A synergistically upregulated their expression in pre-osteoblasts.

The upregulations of VEGF-A and TGF-β1 induced osteogenic genes (e.g., BSP, OPN, and COL1a1) expressions in the non-viral CRISPRa system-engineered pre-osteoblast, which was consistent with other reported works that utilized gene transfer approaches to promote osteogenesis [45]. The induction of BSP, OPN, and COL1a1 kept longer time than those of VEGF-A and TGF-β1, which may be due to the autocrine and paracrine effect induced by the VEGF-A and TGF-β1 [46]. The ALP activity and matrix mineralization are two “gold standards” to evaluate the osteogenesis ability. The inhibition of ALP activities in the CRISPRa treatments at 7 ​d and 14 ​d after transfection may be due to the increased TGF-β1 level. It was reported that the increased TGF-β1 expression abolished the BMP-2-mediated osteogenic gene expression and ALP activity [47]. However, the ALP activity recovered at 28 ​d after transfection, and the combined activation of VEGF-A and TGF-β1 showed the greatest ALP activity and mineralized nodule formation. These results demonstrate that the combined activation of TGF-β1 and VEGF-A synergistically enhanced osteogenesis. In addition, it was reported that the osteogenic effect of TGF-β1 was related with its concentration [48]. The CRISPRa system-mediated TGF-β1 activation using PND polymers increases the expressions of BSP, OPN and COL1a1 genes, which is inconsistent with previous report that the TGF-β1 expression plasmids delivered by commercial transfection reagent lipofectamine inhibited the expressions of ALP, OC, and COL-II genes. This discrepancy may be due to the difference of transfection efficiency (75% vs 24.5%) of PND polymer and lipofectamine, resulting in different TGF-β1 production. However, it was reported that the high amount of exogenous TGF-β1 (5 ​ng/mL) inhibited the bone osteogenesis [49]. In this study, we observe the inductive effects of VEGF-A and TGF-β1 on BSP, OPN, COL1a1, ALP, and mineralized nodule formation. Although the inducing effect of DNA molecules-based non-viral CRISPRa system did not exceed 14 ​d, which may be due to the degradation of DNA by enzyme, it can effectively regulate cell life [50]. The viral vectors (e.g., adeno-associated virus, retrovirus, and baculovirus)-based CRISPRa systems activated osteogenic genes expression for at least 14 days, but they may cause uncontrolled chromosomal integration and immunogenicity [[51], [52], [53]].

To implant the CRISPRa-engineered cells into the lesion site, the injectable hydrogels with a dual-crosslinking structure were used. The combination of the slow-reacting aldehyde-oxyamine conjugation and fast-reacting N_3_-DBCO conjugation is favorable for optimizing mechanical parameters, gelation time, morphology, and biodegradability of hydrogels, achieving high cell encapsulation efficiency and tunable cells release. The traditional gel preparing approaches based on amide formation, radical polymerization, and Michael addition, have a gelation rate that is too slow to encapsulate most cells before they diffuse away from the injury area. In addition, these approaches often involve the use of initiators or precursors which can be toxic to bone due to their reaction with cellular components [54]. By contrast, in this study, the combination of fast-reacting SPAAC and low-reacting hydrozone linkage favors both cell encapsulation by tuning gelation time and cell expansion by the breakage of pH-liable hydrozone linkage under the acidic environment of the injured area [39]. The combination of two types of crosslinking structures provides a useful platform to optimize hydrogels for promoting cell proliferation. The synergistic gene activation of non-viral CRISPRa system and tunable dual-crosslinked hydrogel provide effective approaches to achieve enhanced osteogenesis and angiogenesis. As expect, implantation of the CRISPRa system-engineered MC3T3-E1 cells using dual-crosslinked hydrogel significantly improves the calvarial bone healing by synergistically improving *in vivo* angiogenesis and osteogenesis. The co-activation of TGF-β1 and VEGF-A genes results in more bone formation than any single activation of TGF-β1 or VEGF-A *in vivo*. Consequently, the combination of non-viral CRISPRa system and tunable dual-crosslinked hydrogel substantially improves the bone formation *in vivo*.

## 5 Conclusion

We developed a cationic copolymer PAsp(MTAS-NLS-*co*-DMH) carrying nucleus localizing peptide MTAS-NLS and sponge effect group DMH, showing high cellular uptake, lysosomal escape, and nuclear translocation. This copolymer efficiently delivered CRISPRa pDNA targeting TGF-β1 and VEGF-A to concurrently activate TGF-β1 and VEGF-A genes expressions to synergistically promote osteogenesis. The dual-crosslinked hydrogel was prepared to implant non-viral CRISPRa-engineered pre-osteoblast cells. The hydrogel has tunable mechanical properties, controllable gelation time, and bone regenerative microenvironment-responsive crosslink breakage, which is favor of cell encapsulation and cell expansion in the bone defect site. The *in vivo* studies confirm that the combination of VEGF-A and TGF-β1 activation shows better bone healing than the single use of either VEGF-A or TGF-β1 activation alone. This study paves a potential approach to translate the CRISPRa technology into clinical application.

## Funding

This project was supported by the 10.13039/501100001809National Natural Science Foundation of China (21875289 and 91959118), the Guangdong-Hong Kong-Macao joint innovation Project (2021A0505110011) and 10.13039/501100003453Natural Science Foundation of Guangdong Province (2022A1515011304 and 2020A1515011397).

## Data availability statement

The data that supports the findings of this study are available in the Supplementary Information of this article.

## Declaration of competing interest

The authors declare that they have no known competing financial interests or personal relationships that could have appeared to influence the work reported in this paper.

## References

[1] Roddy E., DeBaun M.R., Daoud-Gray A., Yang Y.P., Gardner M.J. (2018). Treatment of critical-sized bone defects: clinical and tissue engineering perspectives. Eur. J. Orthop. Surg. Traumatol..

[2] Baldwin P., Li D.J., Auston D.A., Mir H.S., Yoon R.S., Koval K.J. (2019 Apr). Autograft, allograft, and bone graft substitutes: clinical evidence and indications for use in the setting of orthopaedic trauma surgery. J. Orthop. Trauma.

[3] Ho-Shui-Ling A., Bolander J., Rustom L.E., Johnson A.W., Luyten F.P., Picart C. (2018). Bone regeneration strategies: engineered scaffolds, bioactive molecules and stem cells current stage and future perspectives. Biomaterials.

[4] Wang W., Yeung K.W.K. (2017 Jun 7). Bone grafts and biomaterials substitutes for bone defect repair: a review. Bioact. Mater..

[5] Peng Y., Li J., Lin H., Tian S., Liu S., Pu F., Zhao L., Ma K., Qing X., Shao Z. (2021). Endogenous repair theory enriches construction strategies for orthopaedic biomaterials: a narrative review. Biomater Transl.

[6] Qasim M., Chae D.S., Lee N.Y. (2020 Mar). Bioengineering strategies for bone and cartilage tissue regeneration using growth factors and stem cells. J. Biomed. Mater. Res..

[7] Diomede F., Marconi G.D., Fonticoli L., Pizzicanella J., Merciaro I., Bramanti P., Mazzon E., Trubiani O. (2020 May 3). Functional relationship between osteogenesis and angiogenesis in tissue regeneration. Int. J. Mol. Sci..

[8] Hu K., Olsen B.R. (2016 Oct). The roles of vascular endothelial growth factor in bone repair and regeneration. Bone.

[9] Wu M., Chen G., Li Y.P. (2016 Apr 26). TGF-β and BMP signaling in osteoblast, skeletal development, and bone formation, homeostasis and disease. Bone Res.

[10] Kuroda S., Sumner D.R., Virdi A.S. (2012). Effects of TGF-β1 and VEGF-A transgenes on the osteogenic potential of bone marrow stromal cells in vitro and in vivo. J. Tissue Eng..

[11] Vo T.N., Kasper F.K., Mikos A.G. (2012). Strategies for controlled delivery of growth factors and cells for bone regeneration. Adv. Drug Deliv. Rev..

[12] Maeder M.L., Linder S.J., Cascio V.M., Fu Y., Ho Q.H., Joung J.K. (2013). CRISPR RNA-guided activation of endogenous human genes. Nat. Methods.

[13] Chavez A., Scheiman J., Vora S., Pruitt B.W., Tuttle M., E P.R.I., Lin S., Kiani S., Guzman C.D., Wiegand D.J., Ter-Ovanesyan D., Braff J.L., Davidsohn N., Housden B.E., Perrimon N., Weiss R., Aach J., Collins J.J., Church G.M. (2015). Highly efficient Cas9-mediated transcriptional programming. Nat. Methods.

[14] Perez-Pinera P., Kocak D.D., Vockley C.M., Adler A.F., Kabadi A.M., Polstein L.R., Thakore P.I., Glass K.A., Ousterout D.G., Leong K.W., Guilak F., Crawford G.E., Reddy T.E., Gersbach C.A. (2013). RNA-guided gene activation by CRISPR-Cas9-based transcription factors. Nat. Methods.

[15] Truong V.A., Hsu M.N., Kieu Nguyen N.T., Lin M.W., Shen C.C., Lin C.Y., Hu Y.C. (2019). CRISPRai for simultaneous gene activation and inhibition to promote stem cell chondrogenesis and calvarial bone regeneration. Nucleic Acids Res..

[16] M. N. Hsu, K. L. Huang, F. J. Yu, P. L. Lai, A. V. Truong, M. W. Lin, N. T. K. Nguyen, C. C. Shen, S. M. Hwang, Y. H. Chang, Y. C. Hu, Coactivation of endogenous Wnt10b and Foxc2 by CRISPR activation enhances BMSC osteogenesis and promotes calvarial bone regeneration, Mol. Ther.. 28(2020), 441-451, doi: 10.1016/j.ymthe.2019.11.029. 10.1016/j.ymthe.2019.11.029 PMC7001053 31882321

[17] Schuh R.S., Poletto É., Pasqualim G., Tavares A.M.V., Meyer F.S., Gonzalez E.A., Giugliani R., Matte U., Teixeira H.F., Baldo G. (2018). In vivo genome editing of mucopolysaccharidosis I mice using the CRISPR/Cas9 system. J. Contr. Release.

[18] Chen Z., Liu F., Chen Y., Liu J., Wang X., Chen A.T., Deng G., Zhang H., Liu J., Hong Z., Zhou J. (2017). Targeted delivery of CRISPR/Cas9-Mediated cancer gene therapy via liposome-templated hydrogel nanoparticles. Adv. Funct. Mater..

[19] He X.Y., Liu B.Y., Peng Y., Zhuo R.X., Cheng S.X. (2019). Multifunctional vector for delivery of genome editing plasmid targeting β-catenin to remodulate cancer cell properties. ACS Appl. Mater. Interfaces.

[20] Qi Y., Song H., Xiao H., Cheng G., Yu B., Xu F.J. (2018). Fluorinated acid-labile branched hydroxyl-rich nanosystems for flexible and robust delivery of plasmids. Small.

[21] Yue H., Zhou X., Cheng M., Xing D. (2018). Graphene oxide-mediated Cas9/sgRNA delivery for efficient genome editing. Nanoscale.

[22] Liu Q., Zhao K., Wang C., Zhang Z., Zheng C., Zhao Y., Zheng Y., Liu C., An Y., Shi L., Kang C., Liu Y. (2019). Multistage delivery nanoparticle facilitates efficient CRISPR/dCas9 activation and tumor growth suppression in vivo. Adv. Sci..

[23] Wu J., Huang J., Kuang S., Chen J., Li X., Chen B., Wang J., Cheng D., Shuai X. (2019). Synergistic MicroRNA therapy in liver fibrotic rat using MRI-visible nanocarrier targeting hepatic stellate cells. Adv. Sci..

[24] Smith T.T., Stephan S.B., Moffett H.F., McKnight L.E., Ji W., Reiman D., Bonagofski E., Wohlfahrt M.E., Pillai S.P.S., Stephan M.T. (2017). In situ programming of leukaemia-specific T cells using synthetic DNA nanocarriers. Nat. Nanotechnol..

[25] Liu M., Zeng X., Ma C., Yi H., Ali Z., Mou X., Li S., Deng Y., He N. (2017). Injectable hydrogels for cartilage and bone tissue engineering. Bone Res.

[26] Naahidi S., Jafari M., Logan M., Wang Y., Yuan Y., Bae H., Dixon B., Chen P. (2017). Biocompatibility of hydrogel-based scaffolds for tissue engineering applications. Biotechnol. Adv..

[27] Guo J.L., Kim Y.S., Xie V.Y., Smith B.T., Watson E., Lam J., Pearce H.A., Engel P.S., Mikos A.G. (2019). Modular, tissue-specific, and biodegradable hydrogel cross-linkers for tissue engineering. Sci. Adv..

[28] Yu F., Cao X., Du J., Wang G., Chen X. (2015). Multifunctional hydrogel with good structure integrity, self-healing, and tissue-adhesive property formed by combining diels-alder click reaction and acylhydrazone bond. ACS Appl. Mater. Interfaces.

[29] Baker A.E.G., Bahlmann L.C., Tam R.Y., Liu J.C., Ganesh A.N., Mitrousis N., Marcellus R., Spears M., Bartlett J.M.S., Cescon D.W., Bader G.D., Shoichet M.S. (2019). Benchmarking to the gold standard: hyaluronan-oxime hydrogels recapitulate xenograft models with in vitro breast cancer spheroid culture. Adv. Mater..

[30] Hozumi T., Kageyama T., Ohta S., Fukuda J., Ito T. (2018). Injectable hydrogel with slow degradability composed of gelatin and hyaluronic acid cross-linked by schiff's base formation. Biomacromolecules.

[31] Jewett J.C., Bertozzi C.R. (2010). Cu-free click cycloaddition reactions in chemical biology. Chem. Soc. Rev..

[32] Peppas N., Hilt J., Khademhosseini A., Langer R. (2006). Hydrogels in biology and medicine: from molecular principles to bionanotechnology. Adv. Mater. - Adv. Mater..

[33] Patntirapong S., Chanruangvanit C., Lavanrattanakul K., Satravaha Y. (2021 Jan). Assessment of bisphosphonate treated-osteoblast behaviors by conventional assays and a simple digital image analysis. Acta Histochem..

[34] Chung Y.M., Simmons K.L., Gutowska A., Jeong B. (2002). Sol-gel transition temperature of PLGA-g-PEG aqueous solutions. Biomacromolecules.

[35] Rico-Llanos G.A., Borrego-González S., Moncayo-Donoso M., Becerra J., Visser R. (2021). Collagen type I biomaterials as scaffolds for bone tissue engineering. Polymers.

[36] Vimalraj S. (2020). Alkaline phosphatase: structure, expression and its function in bone mineralization. Gene.

[37] Kuroda S., Sumner D.R., Virdi A.S. (2012). Effects of TGF-β1 and VEGF-A transgenes on the osteogenic potential of bone marrow stromal cells in vitro and in vivo. J. Tissue Eng..

[38] Tan Y., Huang H., Ayers D.C., Song J. (2018). Modulating viscoelasticity, stiffness, and degradation of synthetic cellular niches via stoichiometric tuning of covalent versus dynamic noncovalent cross-linking. ACS Cent. Sci..

[39] Lu Y., Aimetti A.A., Langer R., Gu Z. (2016). Bioresponsive materials. Nat. Rev. Mater..

[40] Y. You, K. Kobayashi, B. Colak, P. Luo, E. Cozens, L. Fields, K. Suzuki, J. Gautrot, Engineered cell-degradable poly(2-alkyl-2-oxazoline) hydrogel for epicardial placement of mesenchymal stem cells for myocardial repair, Biomaterials. 269(2021), 120356, doi: 10.1016/j.biomaterials.2020.120356. 10.1016/j.biomaterials.2020.120356 PMC7884911 33189358

[41] Narayanan K., Yen S.K., Dou Q., Padmanabhan P., Sudhaharan T., Ahmed S., Ying J.Y., Selvan S.T. (2013). Mimicking cellular transport mechanism in stem cells through endosomal escape of new peptide-coated quantum dots. Sci. Rep..

[42] Adas G., Koc B., Adas M., Duruksu G., Subasi C., Kemik O., Kemik A., Sakiz D., Kalayci M., Purisa S., Unal S., Karaoz E. (2016). Effects of mesenchymal stem cells and VEGF on liver regeneration following major resection. Langenbeck's Arch. Surg..

[43] Poniatowski Ł.A., Wojdasiewicz P., Gasik R., Szukiewicz D. (2015). Transforming growth factor Beta family: insight into the role of growth factors in regulation of fracture healing biology and potential clinical applications. Mediat. Inflamm..

[44] Shi S., Chan A.G., Mercer S., Eckert G.J., Trippel S.B. (2014). Endogenous versus exogenous growth factor regulation of articular chondrocytes. J. Orthop. Res..

[45] Freitas G.P., Lopes H.B., Souza A.T.P., Gomes M.P.O., Quiles G.K., Gordon J., Tye C., Stein J.L., Stein G.S., Lian J.B., Beloti M.M., Rosa A.L. (2021). Mesenchymal stem cells overexpressing BMP-9 by CRISPR-Cas9 present high in vitro osteogenic potential and enhance in vivo bone formation. Gene Ther..

[46] Hu K., Olsen B.R. (2016). The roles of vascular endothelial growth factor in bone repair and regeneration. Bone.

[47] Ehnert S., Zhao J., Pscherer S., Freude T., Dooley S., Kolk A., Stöckle U., Nussler A.K., Hube R. (2012). Transforming growth factor β1 inhibits bone morphogenic protein (BMP)-2 and BMP-7 signaling via upregulation of Ski-related novel protein N (SnoN): possible mechanism for the failure of BMP therapy?. BMC Med..

[48] Xu J., Liu J., Gan Y., Dai K., Zhao J., Huang M., Huang Y., Zhuang Y., Zhang X. (2020). High-dose TGF-β1 impairs mesenchymal stem cell-mediated bone regeneration via Bmp2 inhibition. J. Bone Miner. Res..

[49] Li J., Ge L., Zhao Y., Zhai Y., Rao N., Yuan X., Yang J., Li J., Yu S. (2022 Mar). TGF-β2 and TGF-β1 differentially regulate the odontogenic and osteogenic differentiation of mesenchymal stem cells. Arch. Oral Biol..

[50] Dominguez A.A., Lim W.A., Qi L.S. (2016 Jan). Beyond editing: repurposing CRISPR-Cas9 for precision genome regulation and interrogation. Nat. Rev. Mol. Cell Biol..

[51] Hsu M.N., Huang K.L., Yu F.J., Lai P.L., Truong A.V., Lin M.W., Nguyen N.T.K., Shen C.C., Hwang S.M., Chang Y.H., Hu Y.C. (2020 Feb 5). Coactivation of endogenous Wnt10b and Foxc2 by CRISPR activation enhances BMSC osteogenesis and promotes calvarial bone regeneration. Mol. Ther..

[52] Nguyen N.T.K., Chang Y.H., Truong V.A., Hsu M.N., Pham N.N., Chang C.W., Wu Y.H., Chang Y.H., Li H., Hu Y.C. (2021 Aug). CRISPR activation of long non-coding RNA DANCR promotes bone regeneration. Biomaterials.

[53] Pickar-Oliver A., Gersbach C.A. (2019 Aug). The next generation of CRISPR-Cas technologies and applications. Nat. Rev. Mol. Cell Biol..

[54] Hermann C.D., Wilson D.S., Lawrence K.A., Ning X., Olivares-Navarrete R., Williams J.K., Guldberg R.E., Murthy N., Schwartz Z., Boyan B.D. (2014). Rapidly polymerizing injectable click hydrogel therapy to delay bone growth in a murine re-synostosis model. Biomaterials.

