# Supplementary material for: Non-viral CRISPR activation system targeting VEGF-A and TGF-β1 for enhanced osteogenesis of pre-osteoblasts implanted with dual-crosslinked hydrogel
Source: Mater Today Bio. 2022 Jul 11;16:100356. doi: 10.1016/j.mtbio.2022.100356 (PMC9309523; doi:10.1016/j.mtbio.2022.100356)
Supplement: Multimedia component 1 [file mmc1.docx]

**Supplementary materials**

***1.*** ***Synthesis and characterization of*** ***poly (amino acid) PAsp(******NLS-MTAS-co-DMH)***

***1.1. Synthesis of*** ***N, N-dimethyl-L-histidine***

*L*-histidine (4 g, 25.8 mmol) and sodium triacetoxyborohydride (12.7 g, 60.0 mmol) were dissolved in deionized water (200 mL). After formaldehyde (37 %, 2.9 mL, 38.6 mmol) and acetic acid glacial (1.5 mL) were added into the above solution, the mixed solution was stirred at room temperature for 12 h. The pH of the reaction system was adjusted to 1 with HCl and then extracted with EtOAc (4 x 300 ml). The combined organic phase was washed with brine, dried (anhydrous MgSO4) and filtered. The filtrate was evaporated under vacuum to obtain *N, N*-dimethyl-*L*-histidine.

***1.2. Synthesis of Ba-PBLA***

N-butylamine-termined poly (β-benzyl L-aspartate) (Ba-PBLA) was synthesized by ring-opening polymerization of N-carboxy anhydride of β-benzyl L-aspartate (BLA-NCA) using n-butylamine as an initiator. Briefly, under a nitrogen atmosphere, 3 g of BLA-NCA (12 mmol) was dissolved in 3 mL of anhydrous DMF, and n-butylamine (9.7 μL，0.1 mmol) was dissolved in 50 mL of anhydrous dichloromethane. The two solutions were mixed and kept stirring for 72 h at 35 °C. The mixture was precipitated in excessive cool diethyl ether. The precipitate was washed with diethyl ether, and vacuum-dried to obtain Ba-PBLA. The degree of polymerization for PBLA was 120, as calculated based on the ^1^H-NMR spectrum Fig. S3. (Ba-PBLA: Mn=24.6 kDa, calculated from ^1^H-NMR spectrum; yield: 93%). ^1^H-NMR resonance peaks at 2.6-2.9 ppm (d, -C***H***_2_COOCH_2_-), 4.80 ppm (s, -C***H***CH_2_COO-), 5.0 ppm (s, -C***H***_2_C_6_H_5_), 7.30 ppm (m, -C*H*_2_C_6_***H***_5_).

***1.3. Synthesis of*** ***Ba-PAsp(DEA)***

Firstly, Ba-PBLA was reacted with acetylchloride to synthesize Ba-PBLA-Ac. Under nitrogen atmosphere, 1 g of Ba-PBLA (0.04 mmol) was dissolved in 20 mL of anhydrous CH_2_Cl_2_ in a 50 mL schlenk flask and cooled in an ice water bath. Then, 85 μL of acetylchloride (1.2 mmol) was added. After 11 μL of anhydrous TEA (0.08 mmol) was added dropwise, the solution was stirred for 12 h at room temperature, dialyzed [Molecular Weight Cut-Off (MWCO): 3.5 kDa] against methanol for 1 d and precipitate was filtered. The precipitate was vacuum-dried to obtain Ba-PBLA-Ac. Next, 0.5 g of Ba-PBLA-Ac (0.02 mmol) and 4.88 mL of ethylenediamine (DEA) were dissolved in 30 mL of anhydrous DMSO. The solution was stirred for 24 h at 35 °C and dialyzed (MWCO: 3.5 kDa) against methanol for 48 h to remove excess DEA. The purified solution was rotary-evaporated to remove solvent and vacuum-dried to get polymer PAsp(DEA) (Mn=18.8 kDa, calculated from ^1^H-NMR spectrum; yield: 91%). ^1^H-NMR resonance peaks at 2.6-2.9 ppm (d, -C***H***_2_CONH-), 2.6 ppm (s, -CONHCH_2_C***H***_2_NH_2_), 2.85 ppm (s, -CONHC***H***_2_CH_2_NH_2_), 4.60 ppm (s, -C***H***CH_2_CONH-).

***1.4. Synthesis of*** ***PAsp(DBCO-co-DMH)***

PAsp(DEA) (0.3 g, 0.016 mmol) was dissolved in 5 mL of DMF and mixed with the solution of dibenzocyclooctyne-N-hydroxysuccinimidyl ester (DBCO-NHS, 19.3 mg, 0.048 mmol) in 2 mL of DMF. After being stirred for 24 h at room temperature, 1.83 g of *N, N*-dimethyl-histidine (10 mmol) was reacted with 1-ethyl-3-(3-dimethylaminopropyl) carbodiimide hydrochloride (2.1 g, 11 mmol) and N-hydroxysuccinimide (1.26 g, 11 mmol) in 10 mL of DMF for 2h and the solution was added to the above solution of PAsp(DEA). The mixed solution was stirred for 24 h at 35 °C and dialyzed (MWCO: 3.5 kDa) against methanol for 48 h. The purified solution was rotary-evaporated to remove solvent and vacuum-dried to get polymer PAsp(DBCO-*co*-DMH) (Mn = 38.5 kDa, calculated from ^1^H-NMR spectrum; yield: 87%). ^1^H-NMR resonance peaks at 2.4-2.9 ppm [s, -CHC***H***_2_CONH- and -N(C***H***_3_)_2_], 3.0-3.4 ppm (-HNC***H***_2_C***H***_2_NH-), 4.6 ppm (s, -C***H***CH_2_CONH-), 7.0 ppm (s, -HNC***H*** = C-), 7.2 ppm (m, Ar), 7.8 ppm (s, -HNC***H*** = N-).

***1.5. Synthesis of*** ***Ba-PAsp(NLS-MTAS-co-DMH)***

PAsp(DBCO-*co*-DMH) (100 mg, 0.0026 mmol) was dissolved in 2 mL of DMF, and azide-termined NLS-MTAS peptide (139 mg, 0.026 mmol) dissolved in 1mL of DMF was added under nitrogen atmosphere. After being stirred at room temperature for 24 h, the solution was dialyzed (MWCO: 14 kDa) against deionized water for 2 d, and freeze-dried to obtain PAsp(NLS-MTAS-*co*-DMH). (Mn=54.6 kDa, calculated from ^1^H-NMR spectrum in Fig. 5; yield: 85%). ^1^H-NMR resonance peaks at 2.5-2.9 ppm [s, -CHC***H***_2_CONH- and -N(C***H***_3_)_2_], 3.0-3.4 ppm (-HNC***H***_2_C***H***_2_NH-), 4.7 ppm (s, -C***H***CH_2_CONH-), 7.0-7.2 ppm (m, -HNC***H*** = C- and Ar), 8.2 ppm (s, -HNC***H*** = N-), new peaks at 0.8-2.0 ppm and 4.21 ppm assigned to NLS-MTAS peptide.

***1.6. Serum stability assay***

To evaluate the serum stability of nanoparticles used in the biological tests, PND/pDNA-NPs was incubated at 37°C in alpha-MEM medium supplemented with 10% fetal bovine serum (FBS). The changes of size and zeta potential of nanodrug was monitored up to 48 h using DLS at 25°C.

***2. Synthesis and characterization of dual-crosslinked hydrogel***

***2.1. Synthesis of HA****-****CHO, DBCO****-****PEG, and N_3_****-****PEG***

The characteristic resonance peaks of HA scaffold (-CH at 3.6-3.8 ppm) and -CHO (at 7.8-8.0 ppm) were observed. Composition of HA-CHO was calculated according to the integration value of characteristic peaks attributing to -CH of HA scaffold and -CHO, respectively. Conversion of the aldehyde in HA was 49.1%. Composition of DBCO**-**PEG was calculated according to the integration value of characteristic peaks attributing to -CH_2_ of 8-Arms NH_2_-PEG (at 3.4-3.6 ppm) and -CH of benzene ring in DBCO (at 7.8-7.9 ppm). Conversion of NH_2_ to DBCO in 8-Arms NH_2_-PEG was 33.5%. Composition of N_3_-PEG was calculated according to the integration value of characteristic peaks attributing to -CH_2_ of 8 Arms NH_2_-PEG (at 3.4-3.6 ppm) and -CH of benzene ring in p-Azidobenzoic acid (at 7.75-7.85 ppm). Conversion of NH_2_ to N_3_ in 8-Arms NH_2_-PEG was 37.9%.

***2.2. Swelling property of hydrogels***

The hydrogel was freeze-dried under vacuum for 48 h. The lyophilized sample was weighed and then immersed in PBS (pH 7.4). At different pre-set time intervals, the samples were taken out and weighed after excess water on the surface was absorbed with filter paper. The mass of each sample was measured three times. The swelling ratio of the hydrogel was calculated according to the following formula: Swelling ratio (%) = (Wm-W_0_)/W_0_×100%, where W_0_ and Wm represent the weight of the initial dry hydrogels and wet hydrogels at different time points, respectively.

***2.3. Rheological property of hydrogels***

Rheological property of hydrogel was analyzed on Anton Paar MCR 301 rheometer using parallel plates of 25 mm diameter with the gap at 4 mm. The storage modulus (*G′*) and loss modulus (*G″*) of samples were recorded as a function of frequency and frequency sweep was performed from 0.01 Hz to 2 Hz at 37 °C with strain of 1%. Experiments were performed with three of independent samples.

***2.4.*** ***Cytocompatibility of hydrogels***

To evaluate the cell behaviors in the different hydrogel with/without PND/pDNA-NPs, the live/dead cell staining were performed. Briefly, MC3T3-E1 cells transfected with/without PND/ pDNA-NPs were culture with different hydrogels in 35-mm culture dishes at a density of 1×10^5^ cells per dish and incubated at 37 °C under a humidified atmosphere with 5% CO_2_. After culturing for 1, 7 and 14 days, the cells-loaded hydrogels were stained with Live/Dead kit (Invitrogen, Molecular probes by Life Technologies, USA) at 37 ​°C for 30 ​min. After incubation, samples were washed with PBS and examined by confocal laser scanning microscope (CLSM, Leica SP8, Germany), and the amount of living cells were manually counted using ImageJ.

Cytotoxicity was determined using the Cell Counting Kit-8 (CCK8) (APExBIO Technology LLC, USA). Briefly, MC3T3-E1 cells transfected with/without PND/ pDNA-NPs were culture with different hydrogels in a 96 well plate at a density of 5×10^3^ cells per well. After culturing for 1, 7 and 14 days, 10 μl of the CCK8 reagent was added to each well and quantified the cell metabolism after incubating at 37 °C for 1 h. The absorbance at an OD of 450 nm was measured using a spectrophotometer.

***2.5.*** ***Cellular function of hydrogels***

To evaluate the ability of the different hydrogels for cell differentiation, MC3T3-E1 cells transfected with/without PND/TV-NPs were cultured with different hydrogels in osteogenic medium. After 2 days, MC3T3-E1 cells were collected and subjected to qRT-PCR to detect the expression of TGF-β1 and VEGF-A. Furthermore, after 7 d of osteoinduction, MC3T3-E1 cells were collected and subjected to alkaline phosphatase (ALP) staining and Alizarin Red staining to evaluate cell differentiation.


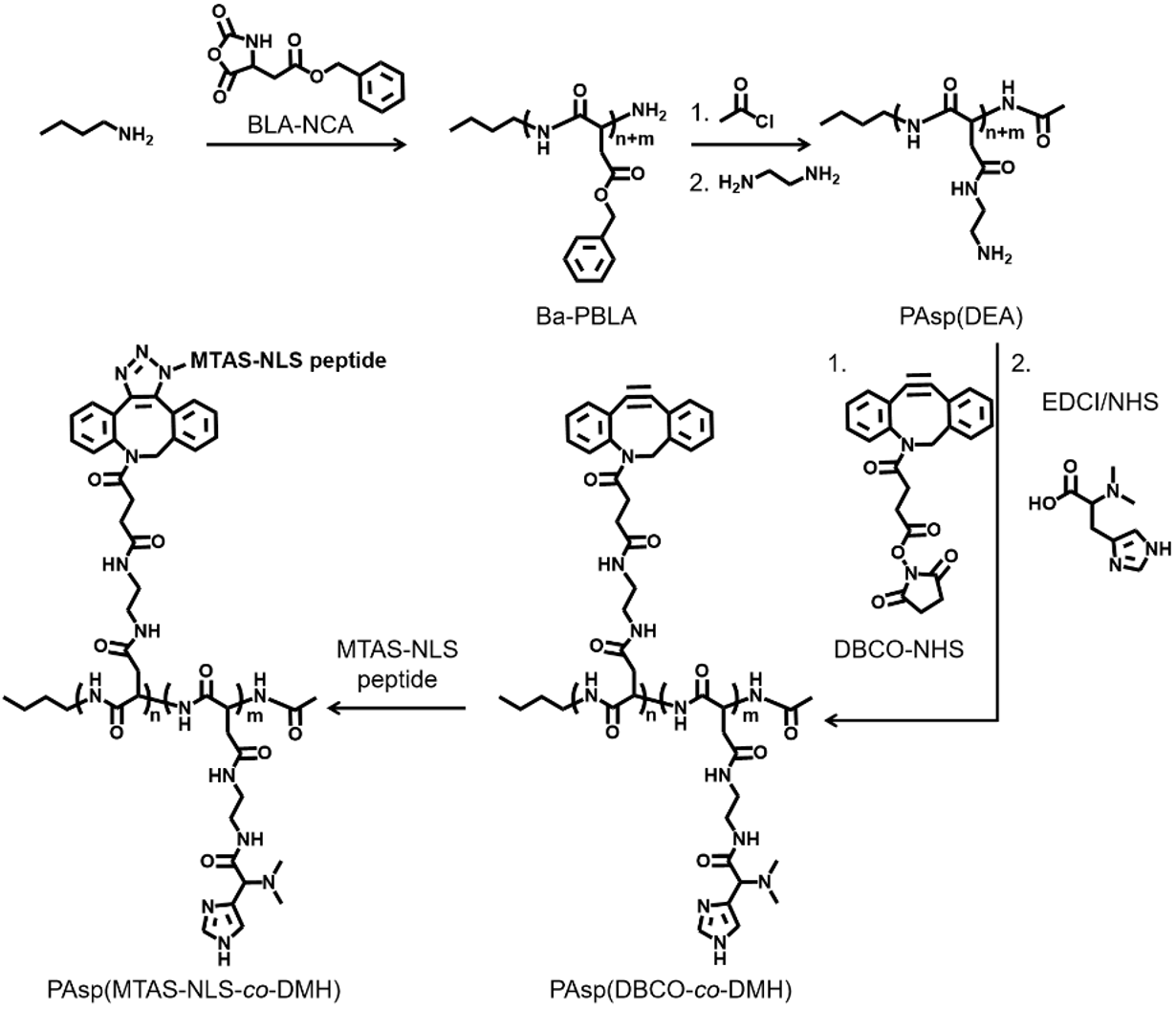


**Scheme S1.** Synthetic routes for the preparation of PAsp (MTAS-NLS-*co*-DMH).


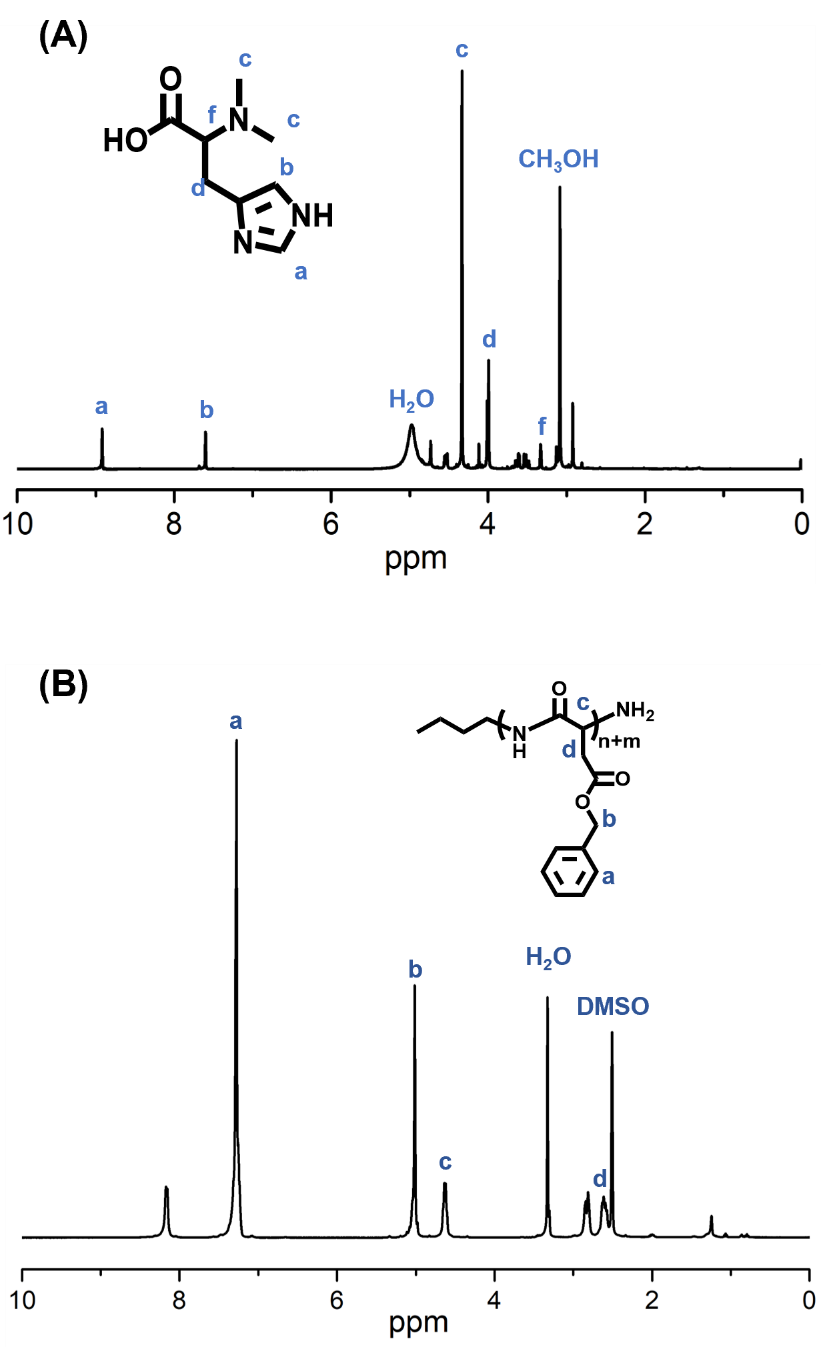


**Fig. S1.** (A) ^1^H-NMR spectrum of Ba-PBLA in DMSO-*d6*. (B) ^1^H-NMR spectrum of Ba-PAsp(DEA) in DMSO-*d6*.


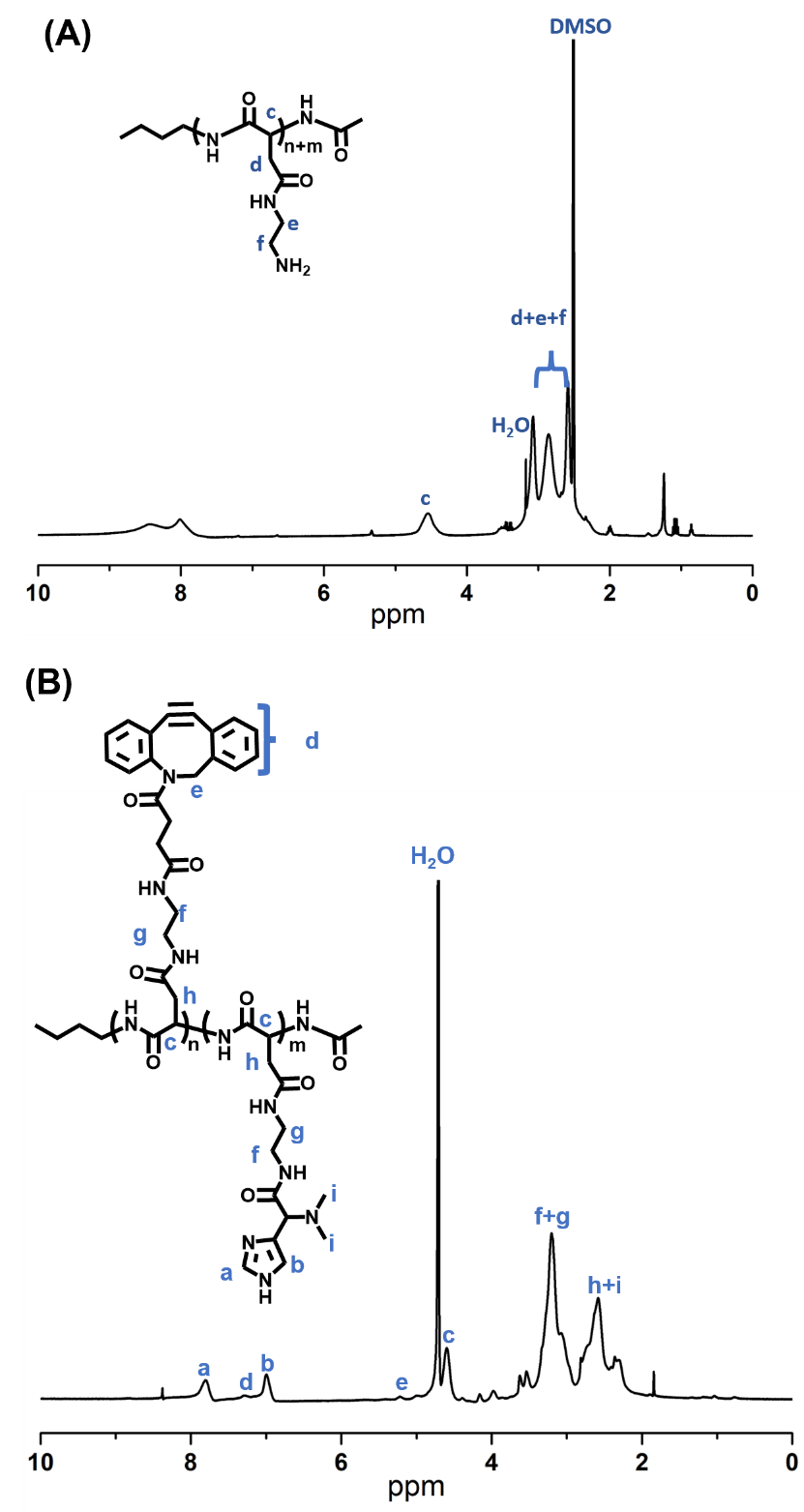


**Fig. S2.** (A) ^1^H-NMR spectrum of PAsp(DBCO-*co*-DMH) in DMSO-*d6*. (B) ^1^H-NMR spectrum of Ba-PAsp(NLS-MTAS-*co*-DMH) in D_2_O.


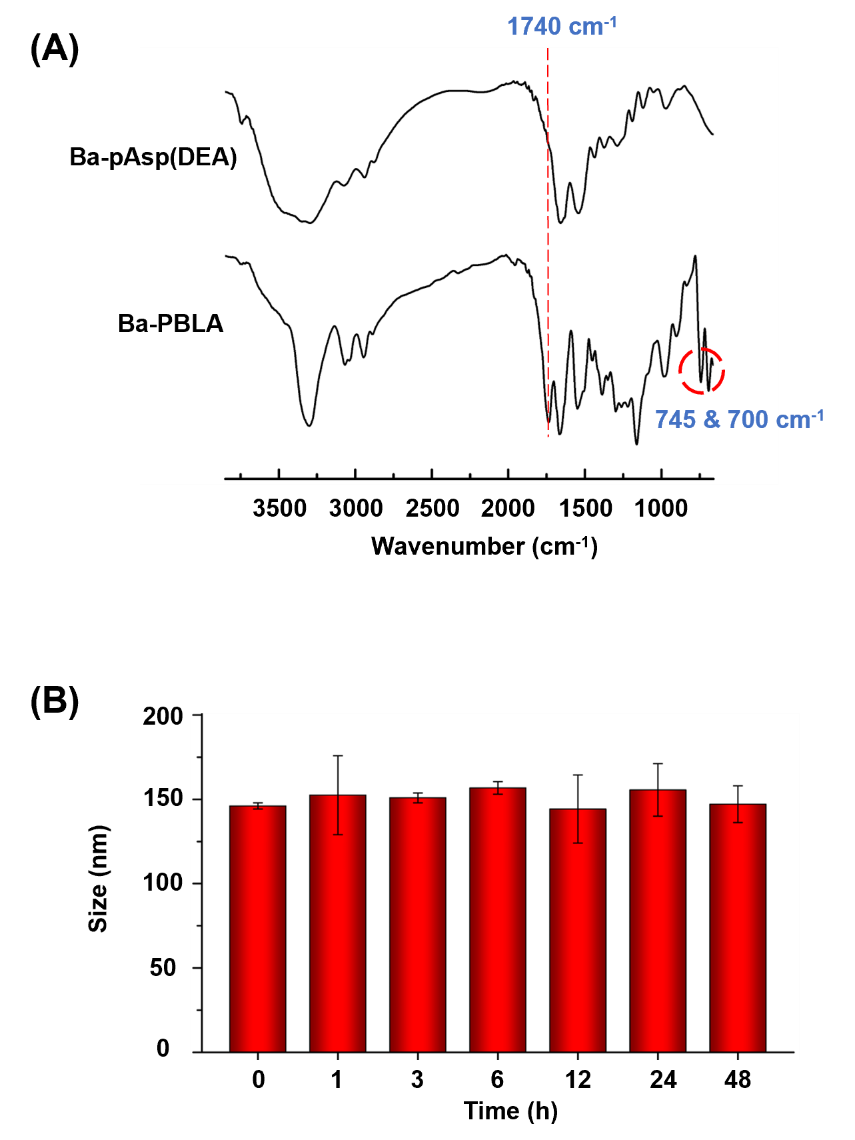


**Fig. S3.** (A) FTIR spectra of Ba-PBLA and Ba-PAsp(DEA). After aminolysis reaction, the characteristic peak of ester at 1740 cm^-1^ (s, *v*_C=O_, ester) and the characteristic peak of benzene at 745 cm^-1^ and 700 cm^-1^ (s, γ_C-H_, benzene) disappeared. (B) Colloidal stability of PND/pDNA-NPs in PBS containing 10% fetal bovine serum (FBS). The sizes of PND/pDNA-NPs were measured through dynamic light scattering assay. Data are presented as the mean ± SD (n = 3).


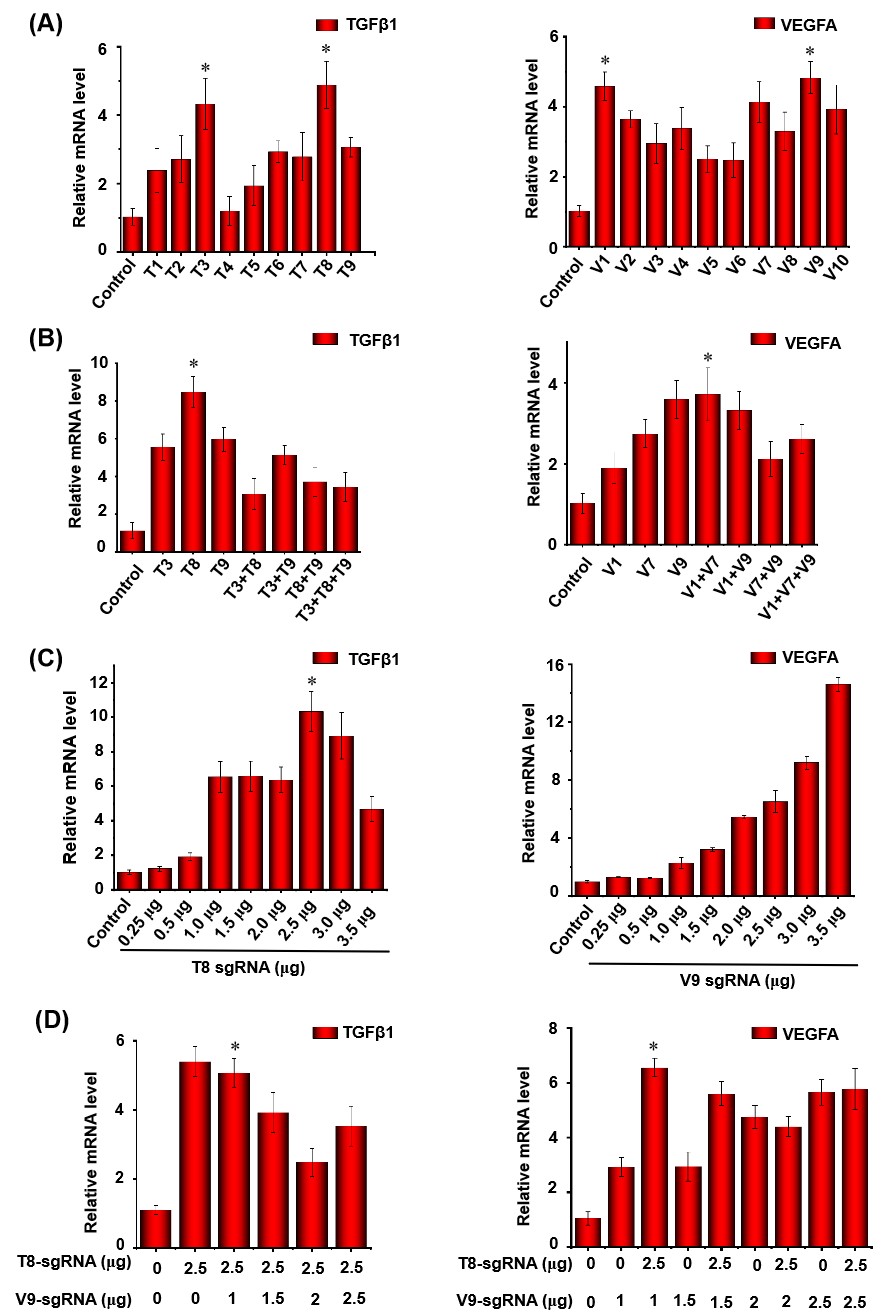


**Fig. S4.** Identification of the CRISPRa system targeting TGF-β1 and VEGF-A. Relative mRNA levels of TGF-β1 and VEGF-A in MC3T3-E1 cells treated with (A) different sgRNA, (B) combined sgRNA, (C) different dosage of sgRNA, and (D) combination of T8 and V9 sgRNAs at different dose. The 9 strands of sgRNA targeting TGF-β1 are designated as T1~T9, and the 10 strands of sgRNA targeting VEGF-A are designated as V1~V10, respectively. The data are mean±SD (n=3). * P＜ 0.05.


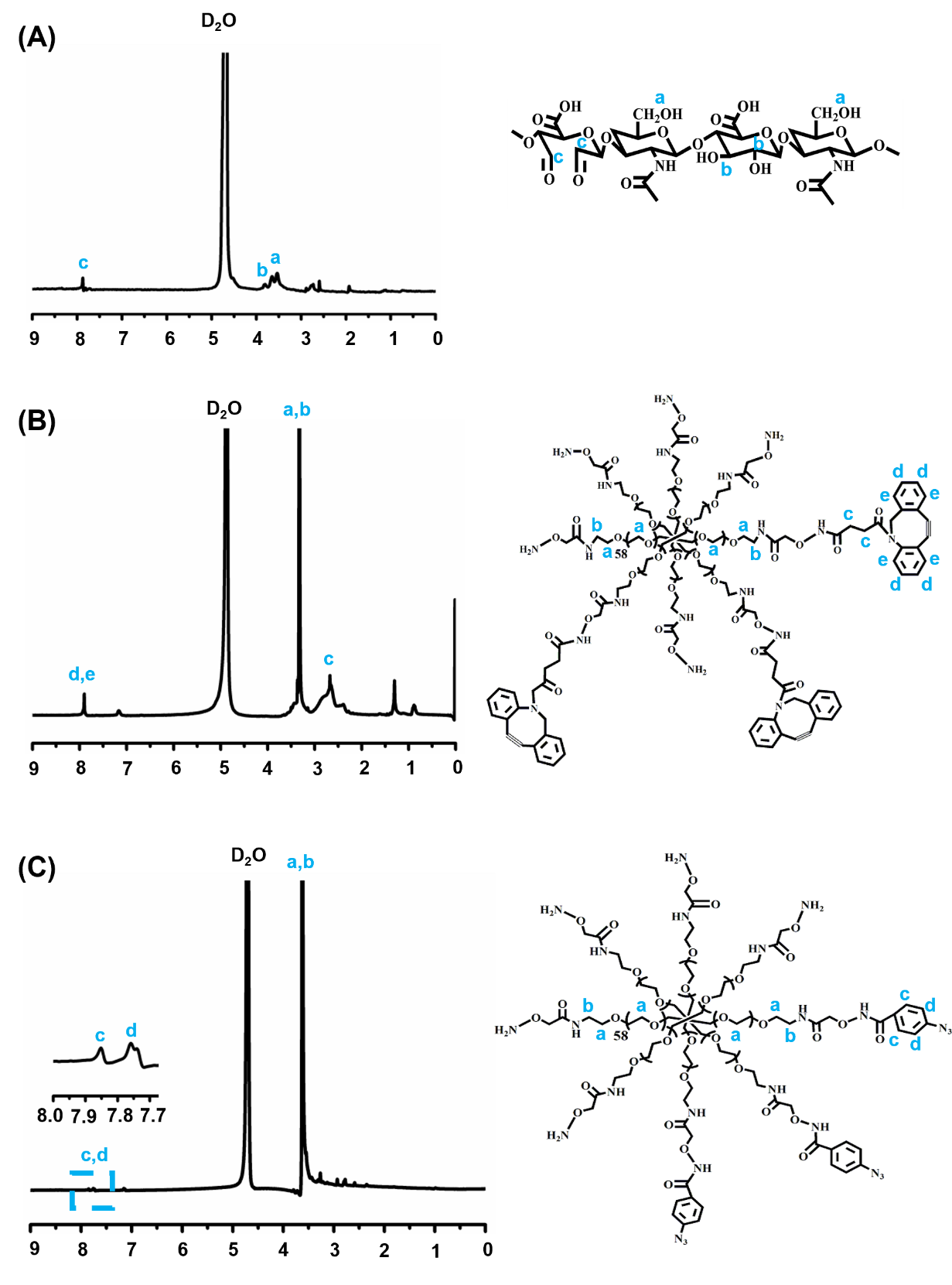


**Fig. S5.** ^1^H-NMR spectrum of (A) HA-CHO, (B) DBCO-PEG and (C) N_3_-PEG.


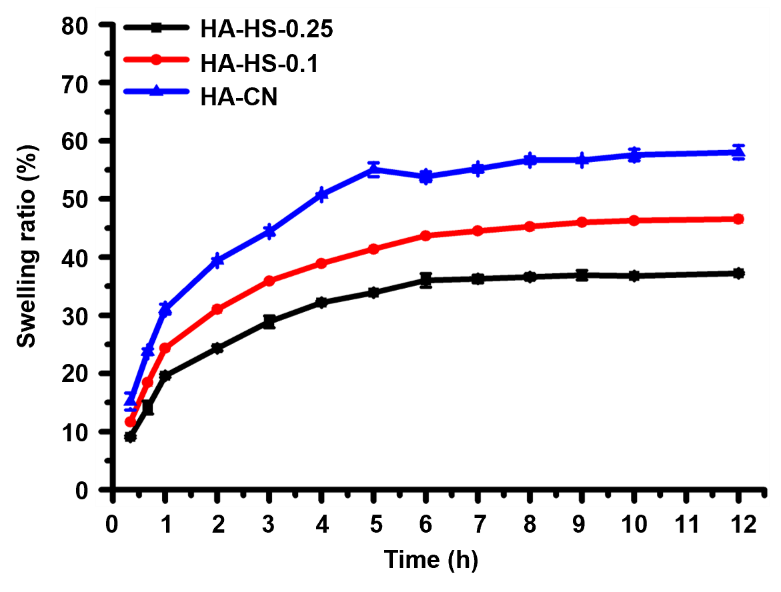


**Fig. S6.** Swelling ratio of hydrogels.


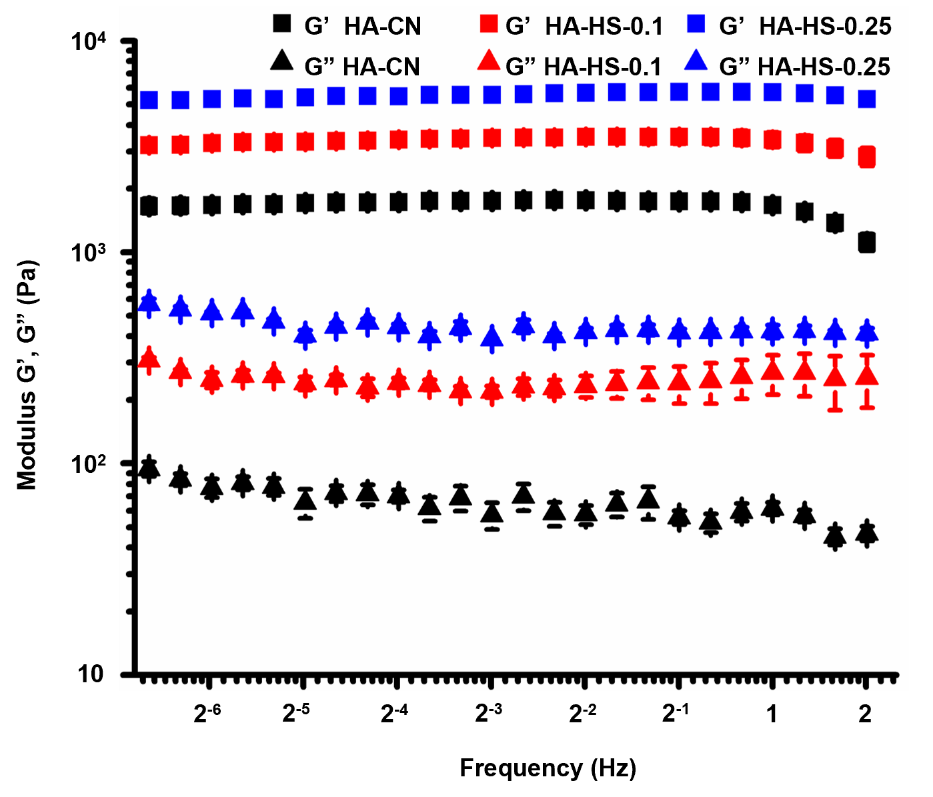


**Fig. S7.** Rheological performance of hydrogels when using oscillation frequency at 37 °C.


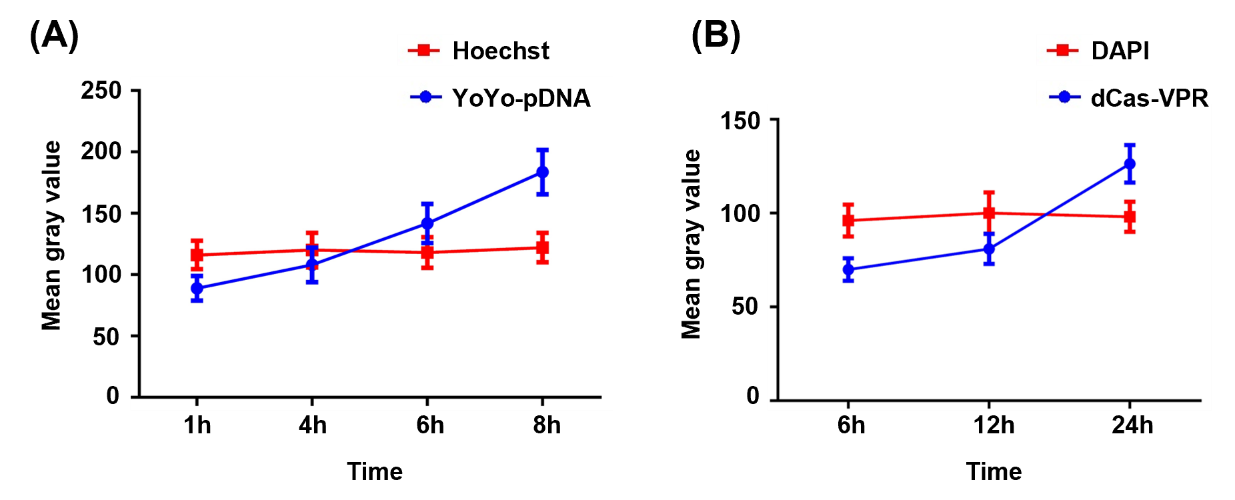


**Fig. S8.** Quantification of fluorescence signal intensities of pDNA and nucleus.


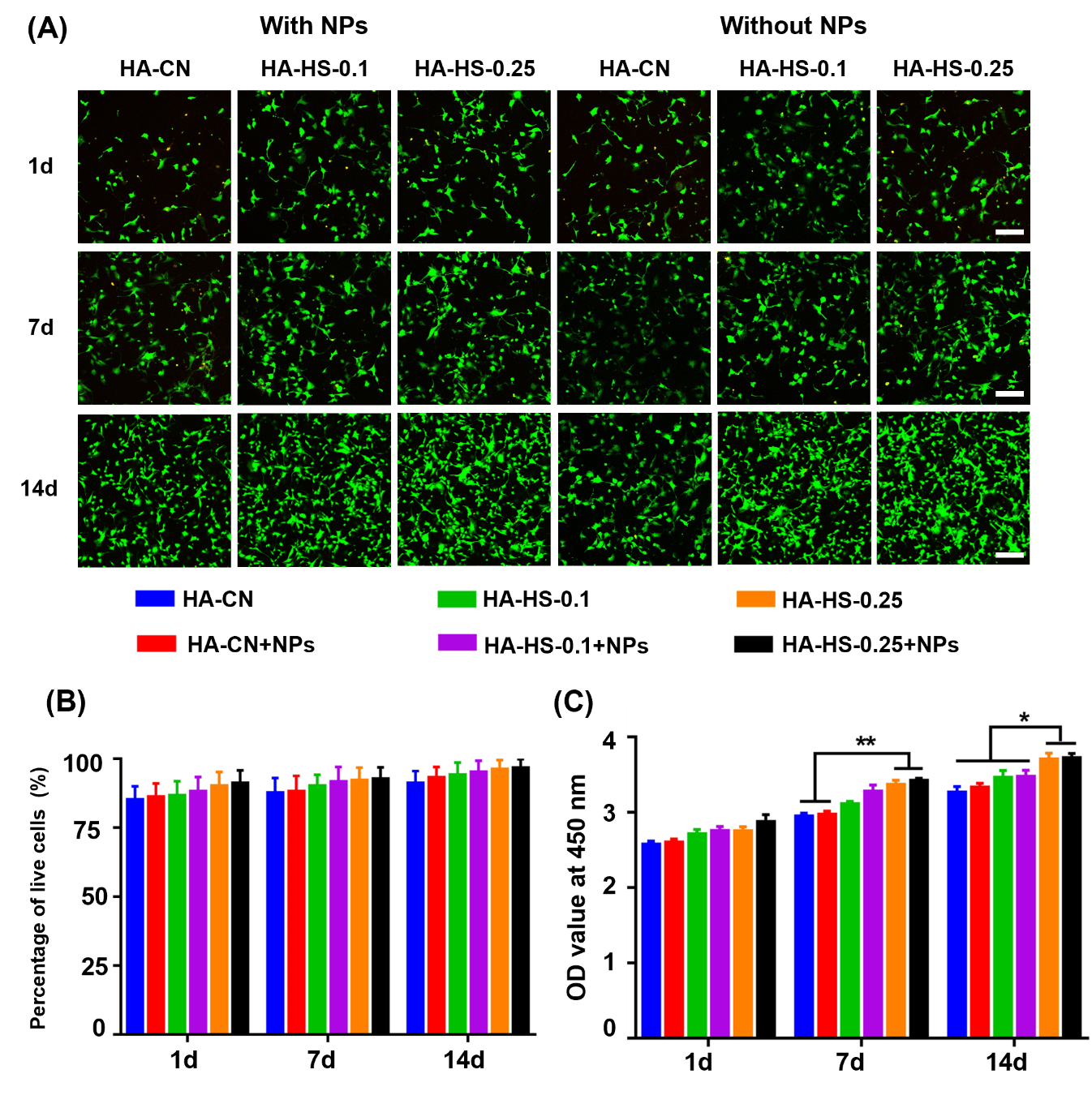


**Fig. S9.** Cytocompatibility of the HA-CN, HA-HS-0.1 and HA-HS-0.25 hydrogels. (A) Live/dead staining of the MC3T3-E1 cells seeded on the HA-CN, HA-HS-0.1 and HA-HS-0.25 hydrogels and cultured for 1, 7, and 14 days. Green: live cells; Red: dead cells. (B) Quantitative analysis of live-dead assay. (C) Cell viability was determined using CCK-8 assays. **p*< 0.05, ***p*< 0.01 versus ratio of the dead cells on HA-HS-0.25, n = 3. Scale bar: 200 μm.


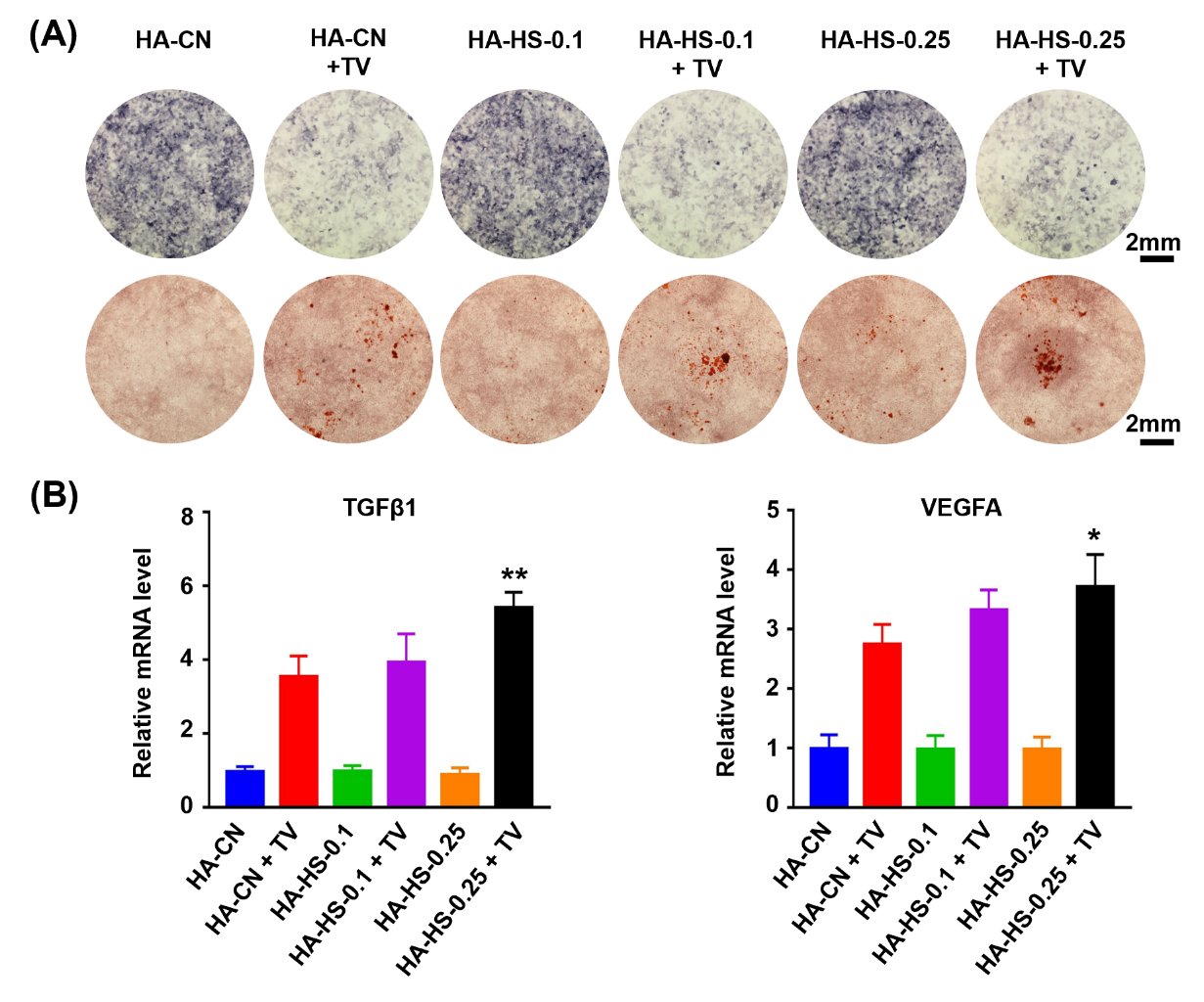


**Fig. S10.** MC3T3-E1 cells encapsulated in the hydrogels with and without PND/TV-NPs. (A) ALP stainling & Alizarin red staining of MC3T3-E1 cells cocultured with different hydrogels for 7 days. (B)Expression of TGFβ1 and VEGFA genes in MC3T3-E1 cells cocultured with different hydrogels measured by quantitative RT-PCR at 2 d after cell encapsulation. The data represent mean ± SD (n=3). **p* < 0.05, ***p* < 0.01.


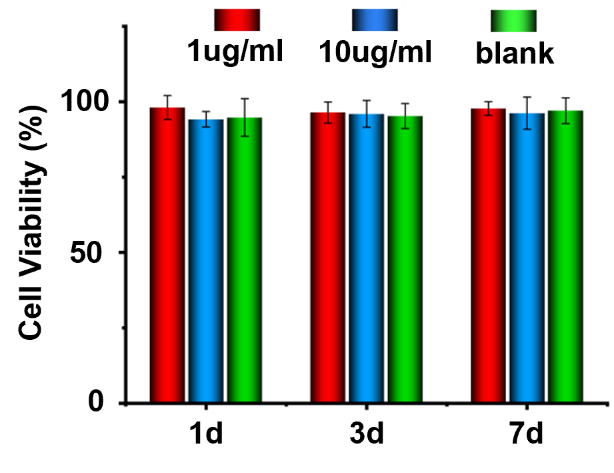


**Fig. S11.** Cytotoxicity of HA-HS-0.25 hydrogels to MC3T3-E1 cells at different concentrations for 1, 3, and 7 days after cell encapsulation, respectively. The data are mean ± SD (n = 3).

**Table S1.** Sequences of sgRNA.

|  |  |  | **Sequences** |
| --- | --- | --- | --- |
| **Target sequences** | **TGF-β1** | **sgRNA1-F** | atccGCTCCTTTGCCGGCTCCCAG |
|  |  | **sgRNA2-F** | atccGGTGACCAACCAAAGCGCCC |
|  |  | **sgRNA3-F** | atccGCGTGGGCGGGCTCCAAGGG |
|  |  | **sgRNA4-F** | atccGGGGGGTCCCTTCAGCCCTG |
|  |  | **sgRNA5-F** | atccGGGACCGGCTGGGGCGGCGG |
|  |  | **sgRNA6-F** | atccGAAGGGTCAAAGGAAAAATT |
|  |  | **sgRNA7-F** | atccGGCCCACTGTTTGGACTGTC |
|  |  | **sgRNA8-F** | atccGGGGTCCCTTCAGCCCTGCG |
|  |  | **sgRNA9-F** | atccGCGGCGGCGGCGGGACCGGC |
|  | **VEGFA** | **sgRNA1-F** | atccGTAAAAAGAGTCTTGCCCAT |
|  |  | **sgRNA2-F** | atccGTGACCCATCCATTCCTGGG |
|  |  | **sgRNA3-F** | atccGGCCATGCCGGGGACATGGG |
|  |  | **sgRNA4-F** | atccGGGCCGGGGAAACACAGGGC |
|  |  | **sgRNA5-F** | atccGAGTGGCCTGGGACAAGAAT |
|  |  | **sgRNA6-F** | atccGTTTACTTCTGGGTGCTAAA |
|  |  | **sgRNA7-F** | atccGGCCCCATCCTTGTCTGACA |
|  |  | **sgRNA8-F** | atccGCAGTCCCTGGAGTAGAATA |
|  |  | **sgRNA9-F** | atccGGCTGCAGCTGGCCAGGATT |
|  |  | **sgRNA10-F** | atccGGAGATGGCTCCTTTGCCAA |

**Table S2.** Primer sequences used for RT-PCR.

| **Real-Time PCR Primers** | **TGF-β1** | **TGF-β1-F** | CTCCCGTGGCTTCTAGTGC |
| --- | --- | --- | --- |
|  |  | **TGF-β1-R** | GCCTTAGTTTGGACAGGATCTG |
|  | **VEGFA** | **VEGFA -F** | TAGAGTACATCTTCAAGCCGTC |
|  |  | **VEGFA-R** | TTTCTTTGGTCTGCATTCACA |
|  | **OPN** | **OPN F** | ACCAGTGATGAGTGTGTGTATT |
|  |  | **OPN R** | TTAGGGTCTAGGACTAGCTTGT |
|  | **BSP** | **BSP F** | AAACACACAGACTTGAGCATTC |
|  |  | **BSP R** | TTAGGGTCTAGGACTAGCTTGT |
|  | **COLⅠ** | **COLⅠ F** | TTTCTTTGGTCTGCATTCACA |
|  |  | **COLⅠ R** | CCATCTTTACCAGGAGAACCAT |
|  | **GAPDH** | **GAPDH-F** | CACCCGCGAGTACAACCTTC |
|  |  | **GAPDH-R** | CCCATACCCACCATCACACC |
